# c-Src kinase is involved in the tyrosine phosphorylation and activity of SLC11A1 in differentiating macrophages

**DOI:** 10.1371/journal.pone.0196230

**Published:** 2018-05-03

**Authors:** Yong Zhong Xu, Thusanth Thuraisingam, Cynthia Kanagaratham, Shao Tao, Danuta Radzioch

**Affiliations:** 1 Department of Human Genetics, McGill University, Montreal, QC, Canada; 2 Research Institute of the McGill University Health Centre, Montreal, QC, Canada; 3 Division of Dermatology, Faculty of Medicine, McGill University, Montreal, QC, Canada; 4 Division of Experimental Medicine, Faculty of Medicine, McGill University, Montreal, QC, Canada; Universita degli Studi di Padova, ITALY

## Abstract

Studies have demonstrated that the solute carrier family 11 member 1 (SLC11A1) is heavily glycosylated and phosphorylated in macrophages. However, the mechanisms of SLC11A1 phosphorylation, and the effects of phosphorylation on SLC11A1 activity remain largely unknown. Here, the tyrosine phosphorylation of SLC11A1 is observed in SLC11A1-expressing U937 cells when differentiated into macrophages by phorbol myristate acetate (PMA). The phosphorylation of SLC11A1 is almost completely blocked by treatment with PP2, a selective inhibitor of Src family kinases. Furthermore, we found that SLC11A1 is a direct substrate for active c-Src kinase and siRNA-mediated knockdown of cellular Src (c-Src) expression results in a significant decrease in tyrosine phosphorylation. We found that PMA induces the interaction of SLC11A1 with c-Src kinase. We demonstrated that SLC11A1 is phosphorylated by Src family kinases at tyrosine 15 and this type of phosphorylation is required for SLC11A1-mediated modulation of NF-κB activation and nitric oxide (NO) production induced by LPS. Our results demonstrate important roles for c-Src tyrosine kinase in phosphorylation and activation of SLC11A1 in macrophages.

## Introduction

Solute carrier family 11 member 1 protein (SLC11A1), formerly known as natural resistance associated macrophage protein 1 (NRAMP1), is an integral membrane protein expressed in cells of the myeloid lineage: monocytes, macrophages, neutrophils and dendritic cells [1,2]. SLC11A1 is present in the membranes of LAMP1 positive late endosomes/lysosomes [3]. Upon phagocytosis of live bacteria or inert particles such as latex beads and zymosan, it is rapidly recruited to the membrane of maturing phagosomes [3–6]. Recruitment of SLC11A1 to the membrane of *Mycobacteria*-containing phagosomes appears to impair the ability of *Mycobacterium* to block phagolysosomal fusion and acidification. It also affects the bacteriostasis or bactericidal activity of phagocytes and the survival of intracellular pathogens. Indeed, it has been reported that SLC11A1-positive mycobacterial phagosomes show enhanced fusion to vacuolar H^**+**^-ATPase-positive vesicles [7] and lysosomes [8,9], increased acidification [7] and enhanced bactericidal activity [5] compared with their SLC11A1-ablated counterparts. SLC11A1 has been shown to function as a proton-dependent transporter for divalent metal cations such as iron and manganese [5,8–11]. It is also involved in iron metabolism [12–15] and exerts pleiotropic effects on macrophage activation [10,16–22]. A study on RAW264.7 murine phagocytes has shown that SLC11A1 deficiency results in reduced formation of nitric oxide (NO), TNF-α and interleukin-6 while interleukin-10 is increased [23]. It has also been shown that an early inflammatory response against bacterial infection occurrs only in SLC11A1^**+/+**^ mice as a result of secretion of pro-inflammatory cytokines such as IFN-γ, TNF-α and MIP-1α [20]. In dendritic cells, SLC11A1 is also expressed in the late endosomal/lysosomal compartments and modulates the expression of cytokines (IL-10 and IL-12) and MHC class II molecules as well as antigen-presentation to T cells [2]. In macrophages, SLC11A1 regulates macrophage functions through inhibition of protein-tyrosine phosphatase activity, in turn modulating the signal pathways associated with NO production and the macrophage response to infection [24]. More recently, SLC11A1 has been reported to be expressed in innate lymphocytes and enhance their activation [25]. The role of SLC11A1 extends beyond its well-established function as a host resistance gene. Recent studies have implicated it in both inflammatory and autoimmune disorders. Meta analysis identified the SLC11A1 locus as playing a role in influencing susceptibility to both infectious and autoimmune diseases [26]. The SLC11A1 gene has also been associated with rheumatoid arthritis [27], type 1 diabetes [28], sarcoidosis [29], Behçet’s syndrome [30], multiple sclerosis [31] and allergic asthma [32]. In addition, through its regulatory effect on the inflammatory cascade, it has been shown to play important roles in tissue regeneration and wound healing [32,33]. However, the exact mechanism of the SLC11A1-mediated effect on inflammation and autoimmunity remains unknown.

Sequence analysis indicates that SLC11A1 protein contains proline-rich motifs (PRMs) that resemble Src homology 3 (SH3) binding domains known to be involved in signal transduction [34–38]. The SH3 domain is a small protein domain of about 60 amino acid residues first identified as a conserved sequence in the non-catalytic part of several cytoplasmic tyrosine kinases such as Abl and Src. It has since been identified in a number of other unrelated protein families such as phospholipases, PI3 kinases, ras GTPase activating proteins, adaptor proteins, CDC24 and CDC25 [36,37]. SH3 domain-containing proteins mediate protein-protein interactions via binding to specific PRMs in their respective target proteins [36–39], which are required for signal transduction, subcellular localization, and cytoskeletal organization in eukaryotic organisms [40–43]. c-Src (or Src) is a well-characterized nonreceptor tyrosine kinase that belongs to a family of eleven related proteins (Src, Lck, Hck, Fyn, Fgr, Yes, Blk, Yrk, Frk, Srm and Lyn) known as Src family kinases (SFKs) [44]. Each member of the SFKs is composed of a series of modular domains that regulate cellular localization (SH4), interaction with binding partners (SH2 and SH3) and enzymatic activity (SH1) [45]. It has been shown that c-Src associates with a variety of growth factor receptors, integrins, and ion channels as well as with several other cellular proteins, leading to the activation of multiple phosphorylation signaling cascades and increased transcription and/or activity of proteins involved in cell growth and proliferation, ion transport, cell motility and invasion [46–50].

Analysis of the amino acid sequence of human SLC11A1 using Netphos 2.0 server [51] and GPS2.1 [52] revealed the presence of two potential tyrosine phosphorylation sites (Y15 and Y38) at its N-terminus. *In vitro* phosphorylation assay have shown that murine Slc11a1 can be phosphorylated at the N-terminal region [53]. However, tyrosine phosphorylation of SLC11A1 has not been reported. In this study, we found that c-Src interacts with SLC11A1 protein during the differentiation of human promyelocytic leukemia cells, U937 and HL-60, into macrophages by PMA. We demonstrated that c-Src tyrosine kinase activity is involved in the phosphorylation of SLC11A1 on tyrosine 15, and that this type of phosphorylation is necessary for SLC11A1-mediated modulation of NF-κB activation and NO production.

## Materials and methods

### Antibodies and reagents

The rabbit anti-SLC11A1 antibody was purchased from Santa Cruz Biotechnology, Inc. (Santa Cruz, CA). The rabbit polyclonal antibody to Src and mouse monoclonal antibody 9E10 directed against the c-Myc protein were purchased from Abcam (Cambridge, MA). Purified recombinant active c-Src, Anti-phospho-Src (Tyr 418) and anti-phosphotyrosine antibody 4G10 were from Upstate (Lake Placid, NY). The mouse anti-actin monoclonal antibody and HRP-conjugated rabbit anti-mouse IgG were from Sigma (Saint Louis, MO). HRP-conjugated goat anti-rabbit IgG was from Cell Signalling (Danvers, MO). Src substrate Sam68 (amino acids 331–443) used for *in vitro* phosphorylation by c-Src kinase was purchased from Santa Cruz Biotechnology. Cell Line Nucleofector^TM^ kit V was purchased from Amaxa (Gaithersburg, MD). siRNA SMARTpool (c-Src) was from Dharmacon (Lafayette, CO). SFK inhibitor PP2 and PP3 were purchased from Calbiochem (San Diego, CA). The QuickChange II XL site-directed mutagenesis kit was purchased from Stratagene (La Jolla, CA). Activated CH-Sepharose 4B was from Amersham Bioscience.

### Plasmids and constructs

The *SLC11A1* gene was amplified by PCR using full-length human *SLC11A1* cDNA (R&D system) as a template. The forward and reverse primers were as follows: forward primer, 5’-GTCGAATTC**GCCACCATG**ACAGGTGACAAG GGTCCCCAAAG-3’ containing an EcoRI site (underlined) and a Kozak sequence (bold type); reverse primer, 5’- ATGGATCCTTA**CAGATCCTCTTC TGAGATGAGTTTTTGTTC**GCCAGAGGTCTCCCCTTTCTGG-3’ containing a BamHI site (underlined) and a sequence encoding myc epitope (bold type). The PCR product was digested with EcoRI and BamHI and inserted in the corresponding sites of plasmid pCB6, yielding a construct pCB6-SLC11A1-Myc with a Myc tag (EQKLISEEDL) at the C-terminal end of SLC11A1. The c-Myc-tagged PRM-deletion mutant, lacking amino acids 21–29, was constructed using QuickChange II XL site-directed mutagenesis kit. Primers used for deletion were 5’-TCCAGCTATGGTTCCATCTCCAGCCAGCAAGCACCTCCCAGAGAGA CC-3’ and 5’-GGTCTCTCTGGGAGGTGCTTGCTGGCTGGAGATGGAACC ATAGCTGGA-3’. Two mutations (P130A and P231A) in the two different PRMs of SLC11A1 as well as the Y15F and Y38F mutants were generated using the QuickChange II XL site-directed mutagenesis kit according to the manufacturer’s instructions. The cDNA encoding wild-type c-Src in the Puse (-) vector (Upstate Biotechonology, Lake Placid, NY) was subcloned into XhoI /BamHI sites of pCB6 vector, yielding a pCB6-c-Src construct. All mutations and cDNA constructs were confirmed by DNA sequencing.

### *In vitro* peptide binding assay

Three peptides were synthesized by NEO BioScience (Cambridge, MA). The PRM peptide II (ISSPTSPTSPGPRQAPPRET), which spans SLC11A1 amino acids 19–38, comprises a PXXPXXPXP motif. The PRM peptide I (GEVCHLY YPKVPRTVLWLTI), which spans SLC11A1 amino acids 122–141, comprises a PXXPR motif. The negative control peptide (IPDTKPGTFSLRKLWAFTG PGFLM), which spans SLC11A1 amino acids 45–68, lacks a PRM. The positive control peptide is Src substrate Sam68 (amino acids 331–443, Santa Cruz). Each peptide was coupled to activated CH-Sepharose 4B according to the manufacture’s instructions. *In vitro* binding of c-Src to peptides was performed as described previously [54]. The bound proteins were eluted by boiling in SDS sample buffer and analyzed by Western blotting using a rabbit polyclonal antibody to c-Src. Prior to peptide competition binding assay, purified c-Src was added 1, 2 or 5 μg of negative peptide, peptide I or peptide II before incubating with the CH-Sepharose 4B coupled with the positive control peptide.

### Cell culture and transfection

The U937-SLC11A1 cell line (stably expressing c-Myc-tagged SLC11A1) [55] was kindly provided by Dr. Phillippe Gros (McGill University, Montreal, QC, Canada), and was cultured in RPMI medium supplemented with 10% heat-inactivated fetal bovine serum (FBS), 20 mM Hepes pH 7.6, 2mM L-Glutamine and 0.5 mg/ml G418. Cell lines were transfected or cotransfected with different kinds of expression vectors using the Cell Line Nucleofector^TM^ kit C according to the manufacturer’s instructions. Briefly, 2×10^6^ log-growth cells were suspended in 100μl of Cell Line Nucleofector^TM^ Solution C and mixed with the appropriate amount of expression vector. The mixture was transferred into an Amaxa-certified cuvette and the cuvette was then inserted into the cuvette holder of the Nucleofector II (Amaxa, Gaithersburg, MD). Transfection was carried out using the program W-001. As for stable transfection, cells were cultured in RPMI containing 20% FBS and allowed to recover for 3 days, followed by G418 (Geneticin, Invitrogen, Burlington, ON) selection for 10 days at a final concentration of 1.0 mg/ml. Clonal sublines were selected by plating on semi-solid methyl cellulose Iscove’s medium (Stem Cell Technologies, Paisley, Scotland) containing 1.0 mg/ml G418. The stably transfected colonies were grown in complete RPMI medium supplemented with 0.5 mg/ml G418, and screened for protein expression.

### Co-immunoprecipitation and immunoblotting

Cultured cells were lysed in 1ml of RIPA buffer (Sigma) containing protease inhibitors. Lysates were incubated at 4°C for 30 min and centrifuged at 10,000g for 20 min. The supernatants were pre-cleared with protein A- or protein G- sepharose for 30 min. Immunoprecipitation was performed overnight at 4°C using a specific antibody. To precipitate the antigen-antibody complex, protein A- or protein G- sepharose was added and incubated for 1 hr at 4°C. After washing with RIPA buffer, the precipitated proteins were eluted by boiling in SDS sample buffer. Immunoprecipitates or equal amount of cell lysates from each cell line were resolved on SDS-PAGE gel, electrophoretically transferred to PVDF membranes and probed with appropriate antibodies. Immunoreactive proteins were detected by the ECL system and quantified by densitometry. For normalization of the signals, the membranes were stripped of antibodies and reprobed with rabbit anti-SLC11A1antibody, and the proteins were quantified as above.

### Immunoprecipitation and *in vitro* kinase assay

After appropriate treatment, cells were lysed in RIPA buffer, pre-cleared and immunoprecipitated with the antibody against c-Src. The immunoprecipitates were washed using a stringent eight-wash protocol following immunoprecipitation, including 4 x RIPA buffer, 2 x low salt buffer(10 mM NaCl, 20 mM Hepes, pH 7.4, 5 mM MnCl_2_) and 2 x kinase reaction buffer (100mM Tris-HCl, pH 7.2, 125mM MgCl_2_, 5mM MnCl_2_, 2mM EGTA, 25μM sodium orthovanadate and 2mM DTT) washes. Then, kinase reaction buffer containing 200 μM ATP and 5ng/ μl substrate Sam68 (amino acids 331–443) was added to the immunoprecipitates, and in vitro kinase reaction was performed at 30°C for 30 minutes. Immunoprecipitates were boiled in SDS sample buffer, separated on SDS-PAGE gel and probed with antibody 4G10 against phosphotyrosine.

### *In vitro* phosphorylation of SLC11A1 protein

Briefly, purified recombinant human SLC11A1 with a GST tag (a.a.1-a.a.178, Novus Biological) was incubated with different concentrations of recombinant active c-Src for 20 min at 30°C in kinase reaction buffer (100mM Tris-HCl Ph 7.2, 125mM MgCl_2_, 5mM MnCl_2_, 2mM EGTA, 250μM sodium orthovanadate and 2mM DTT) with 100μM ATP. The kinase reaction was stopped by adding appropriate 4×SDS sample buffer. Phosphorylation of SCL11A1 was detected by Western blotting using antibody 4G10.

### Electrophoretic mobility shift assay (EMSA)

Cells were treated with PMA for different time points and then the nuclear extracts were prepared as described previously [56]. The NF-kB consensus sequence (5’-AGTTGAGGGGACTTTCCCAGG-3’) or a mutant sequence (5’- AGTTGAGGCGACTTTCCCAGG-3’) was labelled with [γ-^32^P] ATP using DNA 5’ End-Labeling System (Promega, Madison, WI) according to the manufacture’s instruction. For EMSA assay, 10μg of nuclear protein was incubated with ^32^P-labelled NF-κB oligonucleotides (20,000cpm) in 1× EMSA buffer (10 mM Tris-HCl, pH 7.5, 50 mM NaCl, 0.5 mM EDTA, 0.5 mM DTT, 1 mM MgCl_2,_ 4% glycerol v/v, 50 μg/ml poly (dI-dC)) at room temperature for 20 min. DNA-protein complexes were resolved by electrophoresis in native 4% non-denaturing polyacrylamide gels. The gels were transferred to Whatman 3M paper, dried and autoradiographed.

### NF-κB luciferase assay

Cells were transiently transfected with pGL4.32[luc2P/NF-κB-RE/Hygro] vector using the Cell Line Nucleofector^TM^ kit C according to the manufacturer’s instructions. Briefly, 1×10^6^ log-growth cells were suspended in 100μl of Cell Line Nucleofector^TM^ Solution C and mixed with 1.5 μg of pGL4.32[luc2P/NF-κB-RE/Hygro] vector and 0.5 μg pRL-TK vector. Transfection was performed with a Nucleofector^TM^ II device using the program W-001. At 6 hrs after transfection, cells were treated or left untreated with PMA for various time and cell lysates were prepared. Luciferase reporter assays were performed using the Dual-Luciferase® reporter assay system (Promega, Madison, WI), and the luminescence was measured with a Turner Designs model TD-20/20 luminometer. Firefly luciferase activity was normalized to *Renilla* luciferase activity.

### Small RNA interference experiment

U937-SLC11A1 cells were transiently transfected with the siRNA targeting c-Src using the Cell Line Nucleofector^TM^ kit C according to the manufacturer’s instructions. Briefly, 1×10^6^ log-growth cells were suspended in 100μl of Cell Line Nucleofector^TM^ Solution C and mixed with 2μg of c-Src siRNA (20μM in siRNA buffer). The siRNA duplexes used in the experiment were SMARTpool^®^ SRC. Transfection was performed with a Nucleofector^TM^ II device using the program W-001. Transfected cells were cultured for 48 hrs at 37°C in 5%CO_2._

### NO production

Cell were seeded in 12-well plates and were differentiated with PMA (10 ng/ml) for 72 hrs. Then, the medium was replaced with fresh medium containing 0, 20, 40 or 80 ng/ml LPS and the cells were incubated for another 24 hrs. NO production was evaluated by measuring the accumulation of nitrite in the culture medium by the Griess reaction, as previously described [57]

### Flow cytometry

Fluorochrome-conjugated monoclonal antibodies specific for macrophage surface markers were used. Specifically, after differentiation with 10ng/ml PMA for 48 hours, a total of 0.5x10^6^ U937 or U937-SLC11A1 cells were first washed twice with ice-cold 1X PBS to eliminate serum from culture media. The cells were incubated with Fixable Viability Dye eFluor^TM^ 780 (eBioscience^TM^) in ice-cold 1X PBS for 30 minutes at 4°C to label dead cells and washed once with ice-cold 1X PBS containing 0.2% BSA. The cells were subsequently incubated with BUV395-conjugated anti-CD11b (BD Horizon^TM^) and PerCP/Cy5.5-conjugated anti-CD14 (Biolegend^®^) for 30 minutes at 4°C to stain for macrophage surface markers and then washed once with ice-cold 1X PBS containing 0.2% BSA. The cells were resuspended in ice cold 1X PBS containing 0.2% BSA and analyzed on LSRFortessa^TM^ X-20 (BD Biosciences^TM^). The data were analyzed using FlowJo version 10.4.1 (FlowJo^®^).

### Statistics

Data are presented as mean ± S.E. Comparisons between groups were performed using one-way analysis of variance (ANOVA) followed by Tukey's Multiple Comparison Test (TMCT) when ANOVA indicated a statistical significance existed. Significance was established at *P*<0.05.

## Results

### PMA induces tyrosine phosphorylation of SLC11A1 and c-Src expression in U937-SLC11A1 cells

**Fig 1:**
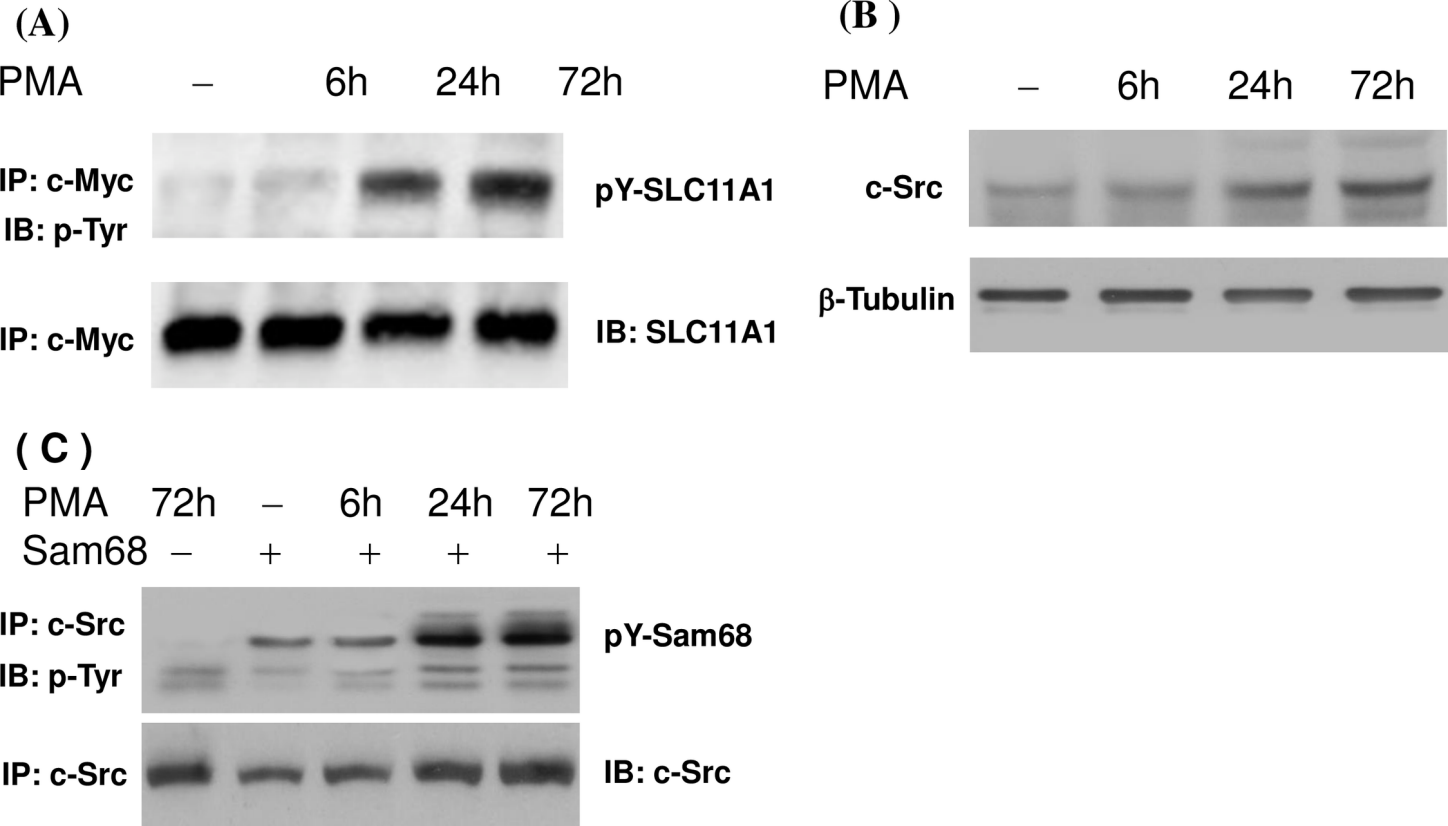
Effect of PMA treatment on the tyrosine phosphorylation of SLC11A1 and Src kinase activity. U937-SLC11A1 cells (stably expressing SLC11A1-c-Myc fusion protein) were left untreated or treated with PMA (10ng/ml) for 6, 24 and 72 hrs. (A) Cell lysates were immunoprecipitated with an anti-c-Myc antibody (9E10). The phosphorylation levels of SLC11A1 were analyzed by immuoblotting with an antibody to phosphotyrosine (4G10). The same blots were reprobed with an anti-SLC11A1 antibody. (B) Cell lysates were separated on SDS-PAGE and the expression of c-Src protein was detected by Western blot analysis. The blots were stripped and re-probed with an antibody against β-tubulin as a loading control. (C) Cell lysates were immunoprecipated with a specific antibody against c-Src. *In vitro* kinase activity assay was performed using Sam68 as a substrate (top panel). The immunoprecipitates were also probed with a specific anti-c-Src antibody (bottom panel).

The U937 cells can be differentiated into macrophages by PMA (S1 Fig). After treatment with PMA for 48 hours, approximate 96% U937 or U937-SLC11A1cells expressed macrophage markers CD11b and CD14. To assess the potential tyrosine phosphorylation of the SLC11A1 in macrophages, U937 –SLC11A1 cell line (stably expressing c-Myc-tagged SLC11A1) was left untreated or treated with PMA for 6 hrs, 24 hrs and 72 hrs. Protein extracts were prepared and subjected to immunoprecipitation using the antibody 9E10. The precipitated SLC11A1 protein complexes were then probed with antibody 4G10 to detect phosphotyrosine residues. As shown in Fig 1A, treatment of U937-SLC11A1 cells with PMA for up to 24 hrs significantly increased the tyrosine phosphorylation level of SLC11A1. PMA-induced phosphorylation was more evident in cells cultured for longer periods. Next, to assess the role of c-Src kinase, we investigated the expression and activity of c-Src kinase in response to PMA treatment. Cells were lysed at various time points after PMA treatment and the total expression of c-Src kinase was monitored by Western blot analysis. The c-Src kinase activity was also analyzed *in vitro* using Sam68 as a phosphorylation substrate for this kinase. As shown in Fig 1B and 1C, PMA treatment significantly increased the expression and activity of c-Src kinase. These results suggest that PMA-induced tyrosine phosphorylation of SLC11A1 may be caused, at least in part, by increased c-Src kinase activity due to increased c-Src kinase expression.

### Src family kinase activities are required for tyrosine phosphorylation of SLC11A1

**Fig 2:**
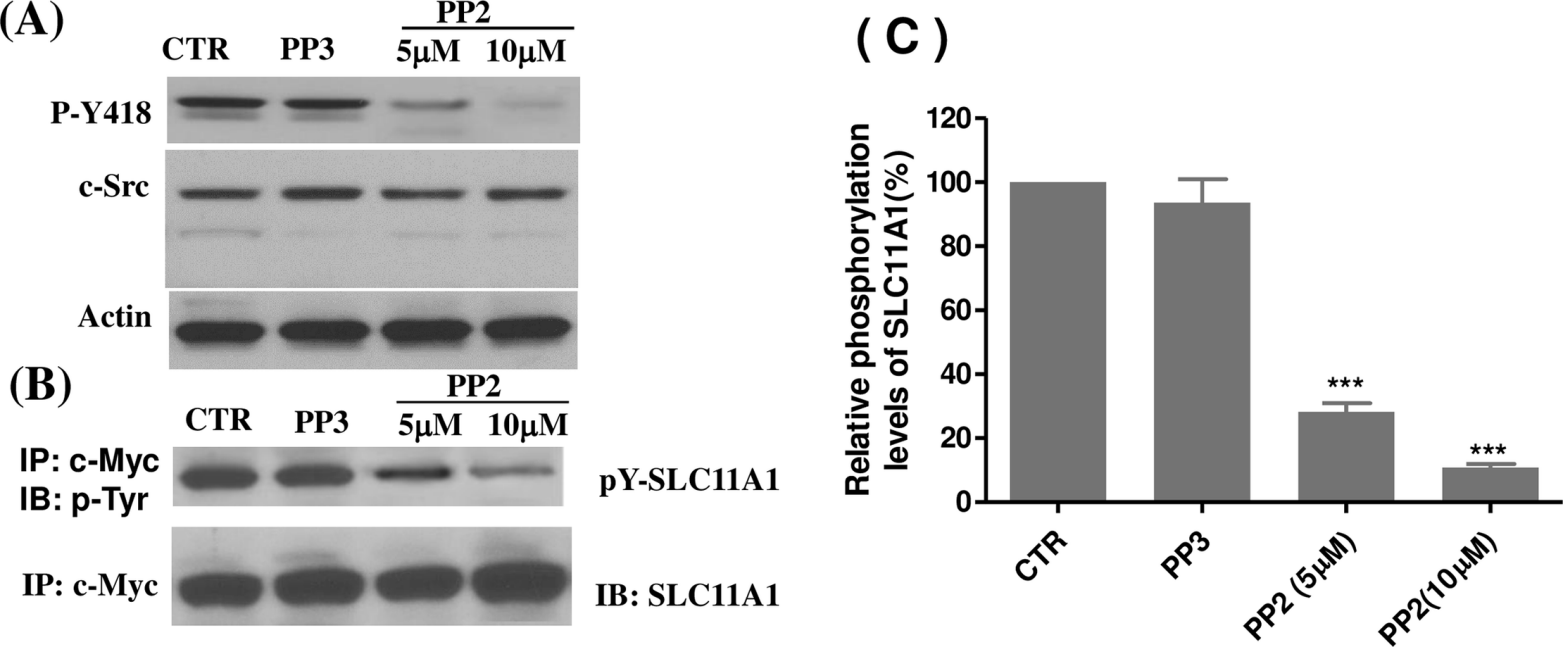
Inhibition of Src family kinase activity blocks tyrosine phosphorylation of SLC11A1. U937-SLC11A1 cells were cultured with PMA (10ng/ml) for 48 hrs and were then left untreated (CTR) or treated with PP2 or PP3 (inactive analogue) for another 24 hours. Cell lysates were prepared. (A) c-Src and active c-Src (pY418) were monitored by Western blotting analysis. (B) Cell lysates were immunoprecipitated with the anti-c-Myc antibody 9E10, and the immunoprecipitates were probed with antibody 4G10 to phosphotyrosine for phosphorylated SLC11A1. The Western blots were stripped and re-probed with an antibody against SLC11A1. (C) The level of SLC11A1 phosphorylation was quantified by densitometry analysis and normalized to the level of total SLC11A1 protein. The relative phosphorylation level of SLC11A1 in untreated control group was set as 100%. The inhibitory effect of PP2 on SLC11A1 phosphorylation was assessed in 3 separate experiments (mean± SE). ***P<0.001, compared with untreated control group.

Tyrosine phosphorylation of SLC11A1 has not been previously reported. In order to determine if Src family kinases are involved in PMA-induced tyrosine phosphorylation of SLC11A1, U937-SLC11A1 cells were first treated with PMA for 2 days, followed by treatment with either the broad spectrum Src family kinase inhibitor PP2, PP3 (an inactive derivative of PP2) or left untreated. Initially, we examined the effect of PP2 on c-Src kinase activity. We used a phospho-specific antibody to detect the active p-c-Src that is phosphorylated on tyrosine 418. This residue, located in the Src tyrosine kinase domain, is autophosphorylated when c-Src is activated and its phosphorylation state is correlated with the kinase activity [58]. As shown in Fig 2A, c-Src phosphorylation on Tyr-418, an indicator of c-Src activation, was clearly inhibited after treatment with 5 μM PP2, and was almost completely inhibited at 10μM. However, PP2 treatment had no effect on the expression of total c-Src. Neither c-Src kinase activation nor c-Src expression was affected by PP3 treatment. Next, to assess whether Src family kinase activity is involved in the tyrosine phosphorylation of SLC11A1, protein extracts from untreated, PP3- or PP2-treated cells were used to detect phosphotyrosine residues in the SLC11A1 protein. As shown in Fig 2B and 2C, tyrosine phosphorylation of SLC11A1 was observed in untreated, differentiated U937-SLC11A1 cells. However, the tyrosine phosphorylation was reduced by approximately 70% in cells treated with 5 μM PP2 and reduced by approximately 90% with 10 μM PP2 (*P*<0.001). The expression of total SLC11A1 protein was not significantly changed with PP2 treatment. PP3, as an inactive analogue of PP2, did not affect the phosphorylation of the SLC11A1 protein. These results demonstrate that Src family kinase activities are required for tyrosine phosphorylation of SLC11A1.

### SLC11A1 protein can be tyrosine phosphorylated by c-Src kinase

**Fig 3:**
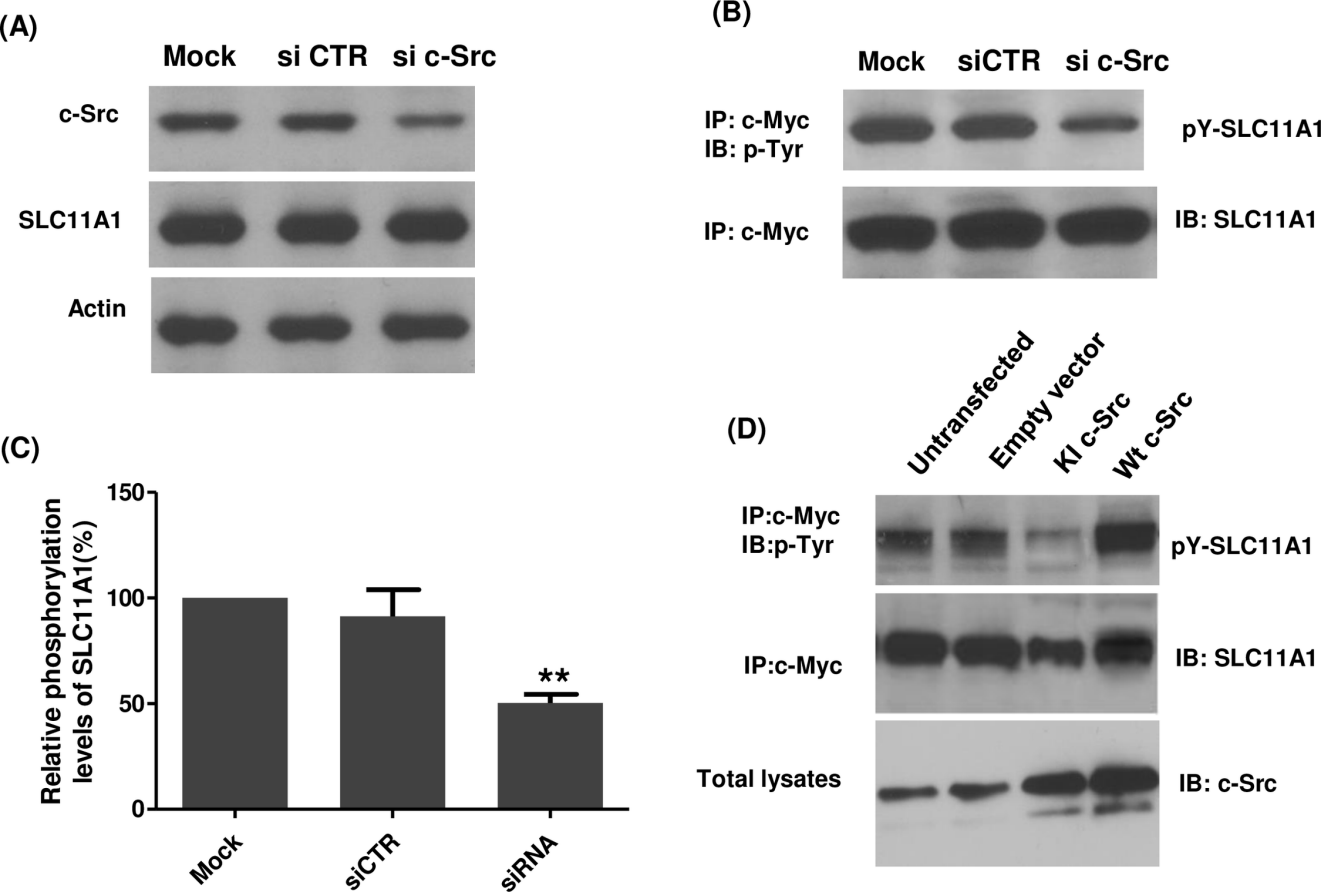
Effects of c-Src knockdown or overexpression of kinase-inactive c-Src on the tyrosine phosphorylation of SLC11A1. U937-SLC11A1 cells were transiently transfected with mock (transfection reagents only), non-targeting scrambled siRNA (siCTR) or c-Src-specific siRNA for 24 h and were then treated with PMA for another 48 hrs. (A) Protein extracts from mock transfected, non-targeting siRNA transfected, or c-Src siRNA transfected cells were prepared, and expression of c-Src, SLC11A1 and β-actin was detected by Western blot analysis using corresponding specific antibodies. (B) Protein extracts were immunoprecipitated with a mouse monoclonal antibody 9E10. Immunoprecipitates were separated on a SDS-PAGE gel, and were probed with antibody 4G10 to phosphotyrosine. The Western blots were stripped and re-probed with an antibody against SLC11A1. (C) The level of SLC11A1 phosphorylation in (B) was quantified by densitometry analysis and normalized to the level of total SLC11A1 protein. The relative phosphorylation level of SLC11A1 in mock-treated cells was set as 100%. Data represented as mean ± S.E.(n = 3) Compared with mock-treated group, **P<0.01. (D) U937-SLC11A1 cells were transiently transfected with empty plasmid (pCB6), plasmid expressing wild type c-Src (pCB6-Src) or expressing kinase-inactive c-Src (pCB6-KI-Src). 24 hrs after transfection, cells were treated with PMA for another 48 hrs and then lysed. Immunoprecipitation and immunoblotting were performed as in (B). Expression of c-Src was also analysed using an anti-c-Src antibody. The illustrated is a representative Western blot analysis showing the effect of c-Src knockdown or overexpression of KI-Src on SLC11A1 phosphorylation.

**Fig 4:**
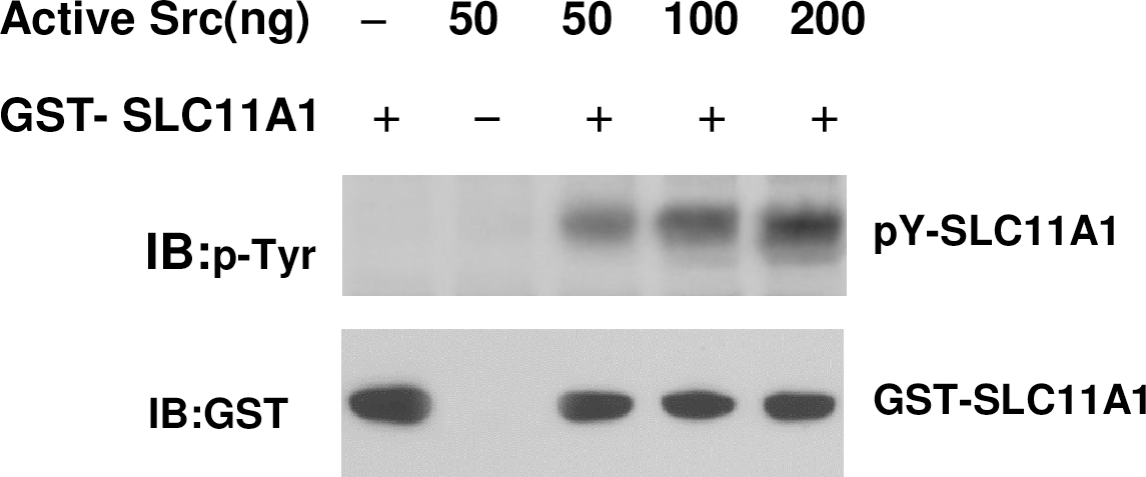
*In vitro* phosphorylation of SLC11A1 by c-Src. A fixed amount of GST-SLC11A1 (500ng, a.a.1-a.a.178) was incubated with increasing amounts (50-200ng) of purified Src, as indicated. Phosphorylation levels of protein were analyzed by immunoblotting with anti-phosphotyrosine antibodies (4G10) (top). The same blots were then reprobed with anti-GST antibodies for GST-SLC11A1 fusion protein as a loading control.

To further determine the level of specificity of tyrosine phosphorylation of SLC11A1 by c-Src kinase activity, we knocked down the c-Src gene using small interfering RNA (siRNA). As shown in Fig 3A, transfection of specific c-Src siRNAs into U937-SLC11A1 cells led to a significant inhibition of c-Src protein expression, with no effect on actin and SLC11A1 expression. Transfection using a non-targeting scrambled siRNA (control siRNA) had no effect on either c-Src or actin levels (Fig 3A). Interestingly, the phosphorylation of SLC11A1 was significantly inhibited in c-Src knockdown cells (*P*<0.01), whereas the expression of total SLC11A1 was not significantly changed (Fig 3B and 3C). Accordingly, to further establish if c-Src activity is required for the phosphorylation of SLC11A1, U973 cells stably expressing SLC11A1 were transiently transfected with vectors to express either kinase-inactive c-Src (K297R, KI c-Src) or Wt c-Src or transfected with empty vectors as a control. As shown in Fig 3D, the tyrosine phosphorylation level of SLC11A1 significantly decreased in cells transfected with KI c-Src but increased in Wt c-Src-transfected cells. Transfection of empty vector did not affect the tyrosine phosphorylation. All these data demonstrate that c-Src kinase activity is involved in the PMA-induced tyrosine phosphorylation of SLC11A1. Finally, to examine whether c-Src phosphorylates SLC11A1 directly, *in vitro* kinase assays were performed using purified c-Src and GST-tagged SLC11A1 protein. As shown in Fig 4, tyrosine phosphorylation of SLCA11A1 increased in a c-Src concentration-dependent manner. Taken together, our results demonstrate that SLC11A1 is directly phosphorylated by c-Src.

### PMA induces the association between Src and SLC11A1 in intact cells

**Fig 5:**
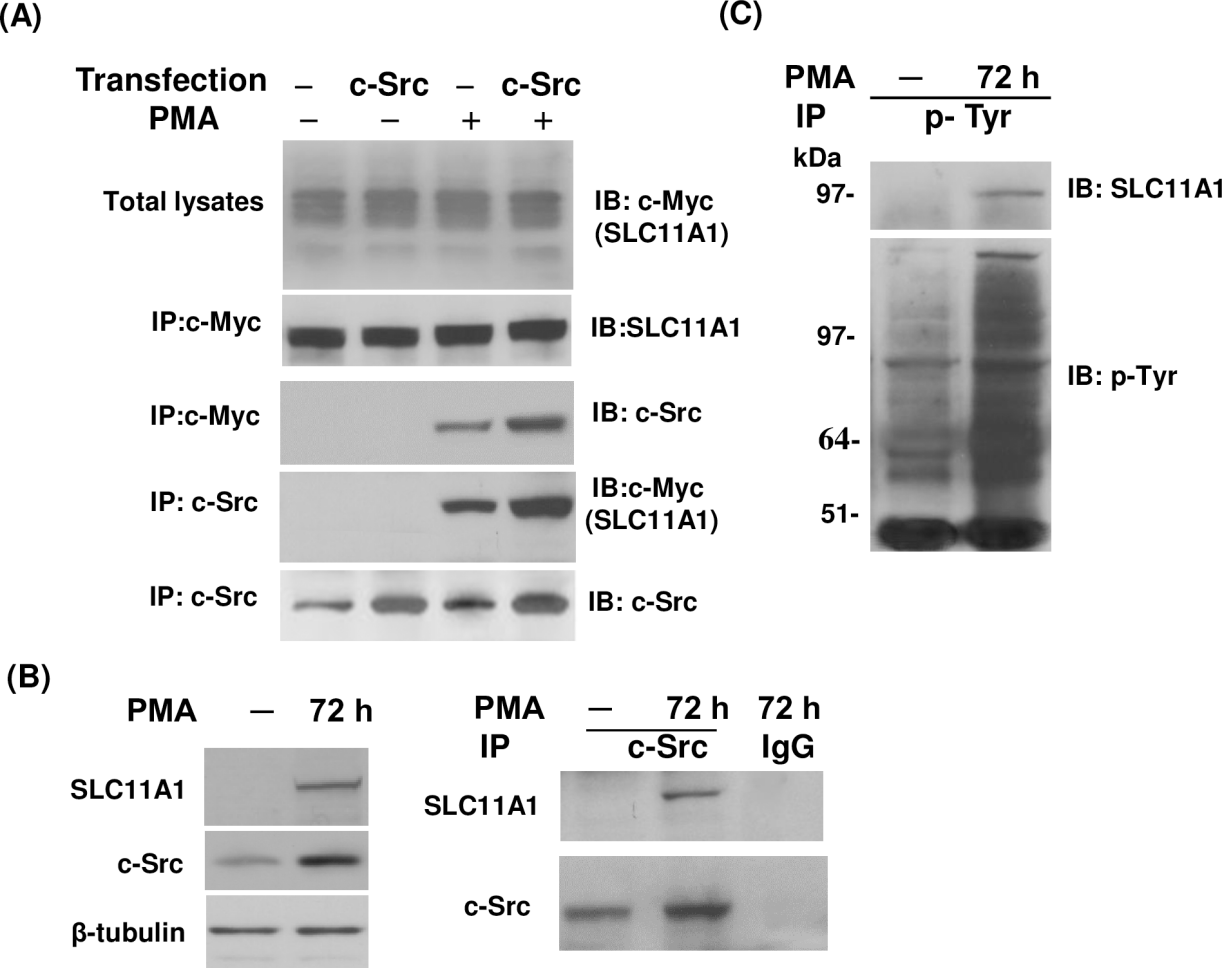
PMA-induced association of SLC11A1 with c-Src in intact cells. U937-SLC11A1 cells were transiently transfected with a vector pCB6-Src to express wild-type c-Src. Untransfected cells were used as a control. Both transfected and untransfected cells were treated with or without PMA (10ng/ml) for 48 hrs as indicated. (A) Cell lysates were prepared and immunoprecipitated with a mouse monoclonal antibody (9E10) directed against the c-Myc tag or anti-c-Src antibodies. Immunoprecipitates were analyzed by Western blot analysis as indicated. (B) HL-60 cells were either treated or untreated with PMA (10ng/ml) for 72 hrs. Cell lysates were prepared and the expression of SLC11A1 and c-Src in response to PMA treatment were detected by using Western blot analysis (left panel). Protein extracts were immunoprecipitated with a c-Src antibody. The immunocomplexes were detected by immunoblotting with an antibody against SLC11A1 and reprobed with an antibody against c-Src (right panel). (C) HL-60 cells were treated with or without PMA (10ng/ml) for 72 hrs. Cell lysates were immunoprecipitated with an anti-phosphotyrosine antibody (4G10). Tyrosine phosphorylation of SLC11A1 was detected by Western blot analysis using a rabbit anti-SLC11A1 antibody. The Western blots were stripped and re-probed with 4G10 for tyrosine-phosphorylated proteins.

To determine whether SLC11A1 and c-Src are associated *in vivo*, U937-SLC11A1 cells were transiently transfected with c-Src and the interaction between SLC11A1 and c-Src was analyzed by co-immunoprecipitation followed by protein immunoblotting with specific antibodies to SLC11A1 and c-Src. As shown in Fig 5A, when using antibody 9E10 to immunoprecipitate the c-Myc tagged SLC11A1 and associated proteins, we were unable to detect the co-precipitation of endogenous and co-expressed c-Src without PMA treatment. However, when cells were treated with PMA for 48 hrs, either endogenous or co-expressed c-Src was found in the complex with SCL11A1. Similarly, when c-Src and associated proteins were immunoprecipitated with a specific antibody against c-Src, we were able to detect the co-precipitation of SLC11A1 with endogenous and co-expressed c-Src in response to PMA treatment. Note that significantly more SLC11A1-c-Src complexes were immunoprecipitated after increasing the c-Src protein levels by transfection (Fig 5A). To determine whether native c-Src and SLC11A1 are also associated under physiological conditions, HL-60 cells were differentiated into macrophages and the association between SLC11A1 and c-Src was also detected using co-immunoprecipitation assay. As shown in Fig 5B, SLC11A1 expression was undetectable in untreated HL-60 cells, as we showed previously [56], and it was also undetectable in c-Src complexes from the untreated HL-60 cells, but it appeared in the c-Src complexes from PMA-treated HL-60 cells. In both untreated and PMA-treated HL-60 cells, c-Src could be detected but there is more after PMA treatment. Our results demonstrate that association of SLC11A1 and c-Src occurs under physiological conditions and does not depend on expression in a heterologous system. To futher determine the tyrosine phosphorylation of native SLC11A1, HL-60 cells were differentiated into macrophages with PMA. Total tyrosine-phosphorylated proteins were immunoprecipitated, and then tyrosine-phosphorylated SLC11A1 were detected by probing with an antibody against SLC11A1. As shown in Fig 5C, tyrosine phosphorylation of SLC11A1 was observed in PMA-differentiated macrophages but not in untreated HL-60 cells. Our results show that endogenous SLC11A1 is also tyrosine-phosphorylated during the PMA-induced differentiation of HL-60 cells towards macrophages.

### Interaction of c-Src with the SLC11A1 via binding to a PXXPR motif

**Fig 6:**
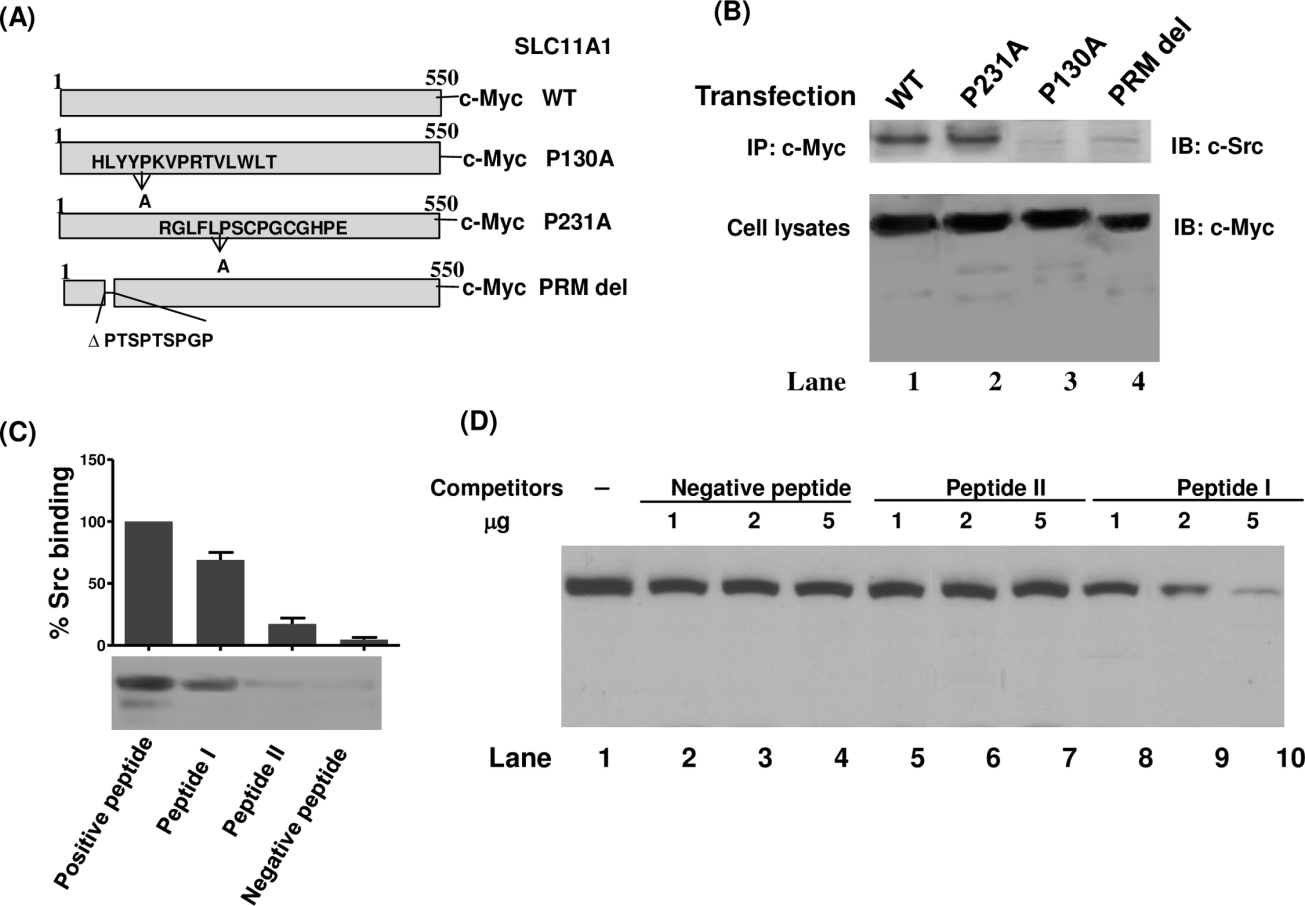
The proline-rich motif of SLC11A1 is required for association with c-Src kinase. (A) Schematic representation of the P130A, P231A and PRM-deletion constructs of SLC11A1. (B) U937 cells were transfected with constructed pCB6 vectors expressing c-Myc-tagged wild-type SLC11A1, P231A, P130A or PRM-deletion mutant together with c-Src expression vector. Transfected cells were treated with PMA for 48 hrs and cell lysates were immunoprecipitated with anti-c-Myc antibody (9E10). The bound proteins were probed with a rabbit polyclonal antibody against c-Src. Expression of c-Myc-tagged wild-type SLC11A1, P231A, P130A or PRM-deletion mutant in transfected cells were also detected by Western blot analysis. (C) *In vitro* binding of c-Src to the PXXP motifs of SLC11A1. The SLC11A1 PRM peptide I (GEVCHLYYPKVPRTVLWLTI), PRM peptide II (ISSPTSPTSPGPRQAPPRE T), the negative control peptide (IPDTKPGTFSLRK LWAFTGPGFLM) and the positive control peptide (Src substrate Sam68), which were described in Materials and Methods, were coupled to activated CH-Sepharose 4B. Binding of these peptides to purified c-Src was detected by immunoblotting with anti-Src antibody. A representative Western blot is shown. (D) Peptide competition binding assay was performed. Purified c-Src was added 1, 2 or 5 μg of negative peptide, peptide I or peptide II before incubating with the CH-Sepharose 4B coupled with the positive control peptide. The illustrated showing the inhibitory effects of different peptides on c-Src binding to the positive peptide.

The SLC11A1 protein contains three PXXP motifs that resemble SH3 binding domains. To test if the PXXP motifs are involved in the interaction of SLC11A1 with c-Src, we constructed three vectors for expression of c-Myc-tagged SLC11A1 mutants as either deletions or amino acid replacements in the three different PXXP motifs of SLC11A1, as depicted in Fig 6A. U937 cells were transfected with these vectors together with the vector expressing c-Src and were then treated with PMA for 48 hrs. Transfected cells were lysed and immunoprecipitated with anti-c-Myc antibody (9E10). The precipitated proteins were separated on an SDS-PAGE gel and probed with a rabbit polyclonal antibody against c-Src. The results showed that the P231A mutatnt, like WT SLC11A1, bound efficiently to a protein band of ~60 kDa corresponding to the expected size of c-Src. However, either the P130A mutation or PRM-deletion greatly diminished the ability of the mutant SLC11A1 protein to bind the c-Src. Binding of the P130A mutant or the PRM-deletion mutant to the c-Src was reduced by 90% and 82%, respectively (Fig 6B, upper panel). Expression of the c-Myc-tagged wild-type SLC11A1, P231A mutant, P130A mutant, or PRM-deletion mutant in transfected cells was also detected by Western blot analysis (Fig 6B, bottom panel). In order to examine whether c-Src binds directly to the PXXP motif of SLC11A1, *in vitro* Src binding assays were performed with synthetic peptides. As shown in Fig 6C, c-Src binds to the Src substrate Sam68 (amino acids 331–443), which was used as a positive control. However, neither a PRM peptide II (ISSPTSPTSPGPRQAPPRET) that comprises a PTSPTSPGP motif nor a negative control peptide (IPDTKPGTFSLRKLWAFTGPGFLM) designed from the N-terminal region that lacks the PTSPTSPGP motif displayed appreciable binding to c-Src. Interestingly, c-Src showd a strong binding affinity to a synthetic PRM peptide I (GEVCHLYYPKVPRTVLWLTI) that matches the region containing a PKVPR motif. To further investigate the specificity of the PKVPR motif, increasing amount of the negative peptide, peptide I and peptide II were used to compete for the PXXPR motif-dependent binding of c-Src to the Src substrate peptide (amino acids 331–443 of Sam68). As shown in Fig 6D, using peptide I we have demonstrated a dose-dependent inhibition of c-Src to the substrate peptide. No inhition or very faint inhibition was observed using either the negative peptide or the peptide II. These results suggest that c-Src kinase may interact with the SLC11A1 protein via binding to the PKVPR motif.

### Phosphorylation of SLC11A1 at Tyr-15 residue by c-Src kinase

**Fig 7:**
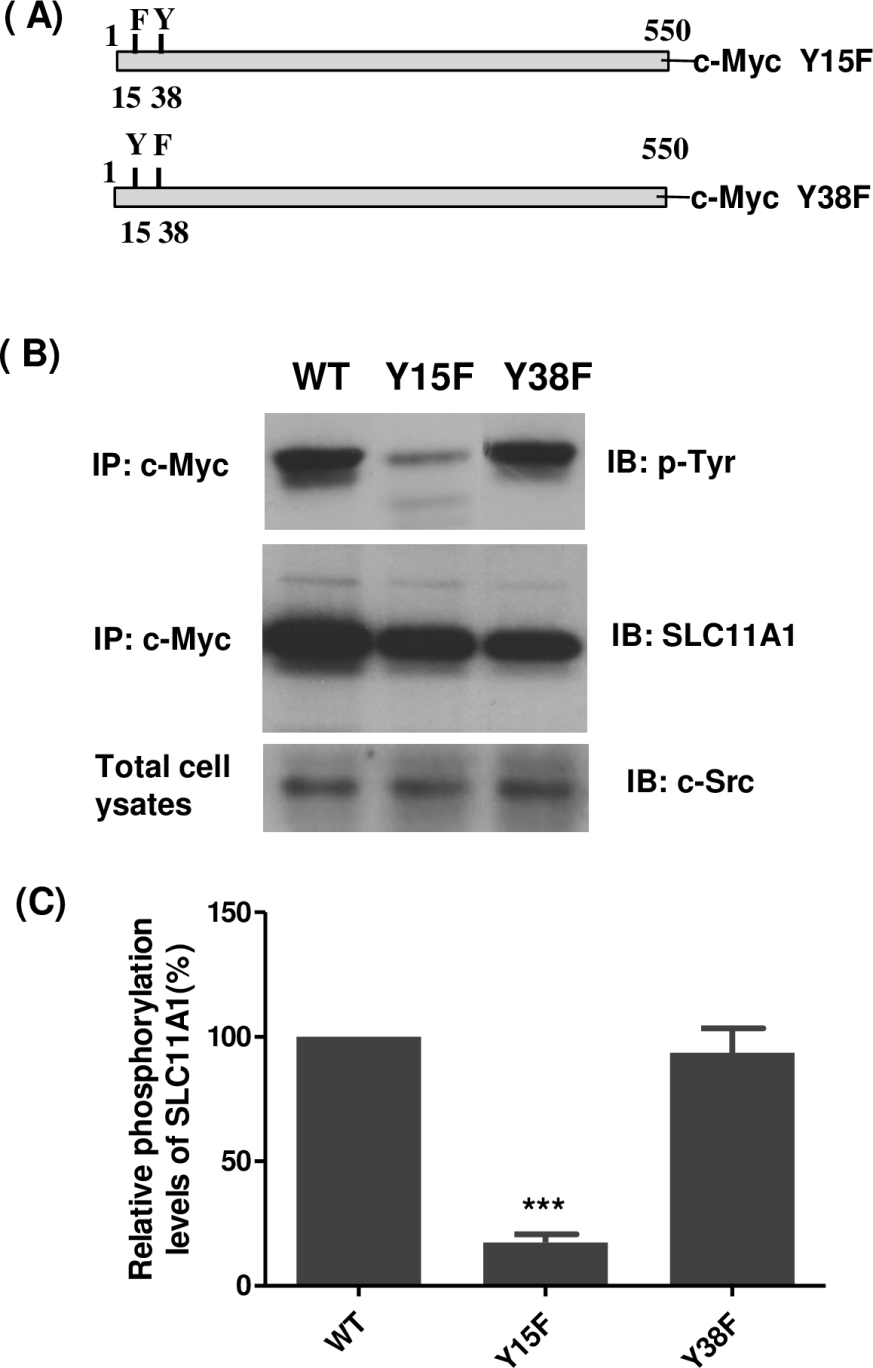
Identification of the tyrosine phosphorylation site of SLC11A1 by c-Src. (A) Site-directed mutagenesis was used to make the Tyr to Phe substitution at position 15 or 38 of SLC11A1. (B) U937 cells stably expressing c-Myc-tagged wild-type or mutated SLC11A1 (Y15F or Y38F) were transiently transfected with a pCB6-Src vector. 24 hours after transfection, cells were treated with PMA for 48 hrs. Cell lysates were prepared and immunoprecipitated with anti-c-Myc antibody (9E10). The tyrosine phosphorylation of SLC11A1was analyzed by immunoblotting with antibody 4G10 to phosphotyrosine (top panel). The Western blots were stripped and re-probed with an antibody against SLC11A1 (middle panel). The expression of c-Src (bottom panel) was also analyzed using anti-Src antibody. (C) The level of SLC11A1 phosphorylation in transfected cells was quantified by densitometry analysis and normalized to the level of total SLC11A1 protein. The relative phosphorylation level of the WT SLC11A1 in U937 cells was set as 100%. Data represented as mean ± S.E.(n = 3). ***P <0.001, compared with WT SLC11A1.

The N-terminal region of the SLC11A1 protein contains two tyrosine residues, Y15 and Y38, which could potentially be phosphorylated by c-Src kinase. To identify the specific phospho-tyrosine residue(s), we generated SLC11A1 constructs bearing tyrosine-to-phenylalanine substitutions at Y15 or Y38 (Fig 7A). c-Myc-tagged wild-type SLC11A1 or substitution mutants were expressed in U937 cells. Transfected cells were treated with PMA for 48 hrs and cell lysates were prepared and subjected to immunoprecipitation with antibody 9E10. The precipitated protein complexes were probed with the anti-phosphotyrosine antibody 4G10 to detect phosphorylated wild-type SLC11A1 or its substitution mutants and their phosphorylation levels were quantitated. As illustrated in Fig 7B and 7C, tyrosine phosphorylation of the Y15F mutant of the SLC11A1 protein was dramatically lower compared to wild-type SLC11A1 (*P*<0.001). Conversely, the phosphorylation level of the Y38F mutant was almost the same as for the wild-type SLC11A1. Overall, these results demonstrate that the SLC11A1 protein is primarily phosphorylated by c-Src kinase on tyrosine 15.

### Tyr-15 phosphorylation is required for SLC11A1-mediated modulation of NO production

**Fig 8:**
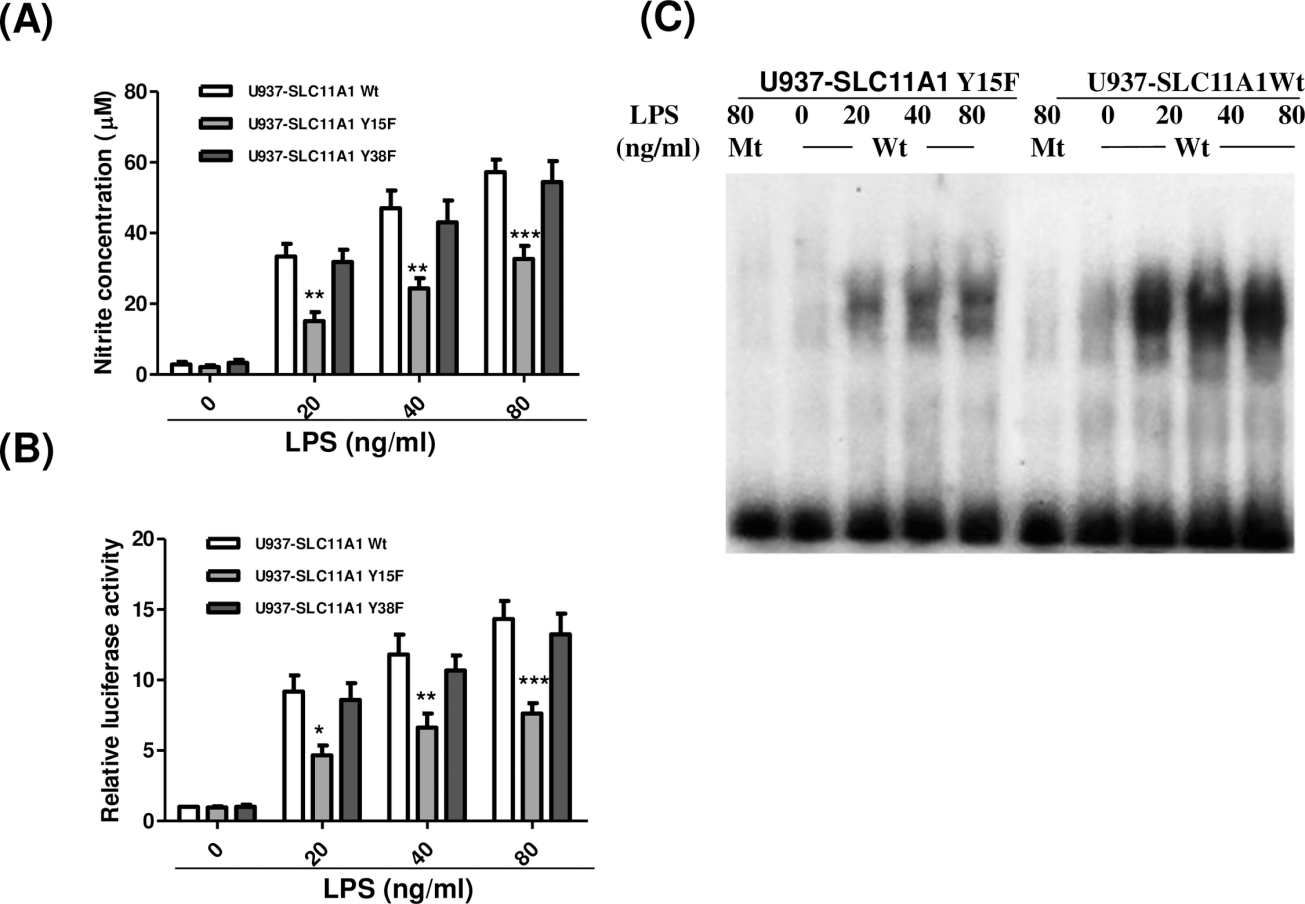
Effects of Tyr-15 to Phe substitution on SLC11A1-mediated NO production and NF-κB activation. U937 cells were stably transfected with constructed pCB6 vectors expressing c-Myc-tagged wild-type (U937-SLC11A1 Wt) or mutated SLC11A1 (U937-SLC11A1Y15F and U937-SLC11A1Y38F). (A) Stably transfected cells were treated with PMA (10ng/ml) for 72hrs. Then, the medium was replaced with fresh medium containing various concentrations of LPS as indicated and the cells were continuously cultured for 24 hrs. Release of NO was measured using Griess reagent (mean ± S.E., n = 4, **P <0.01, *** *P* <0.001). (B) U937-SLC11A1Wt, U937-SLC11A1Y15F and U937-SLC11A1Y38F cells were transiently co-transfected with 1.5 μg of pGL4.32[luc2P/NF-κB-RE/Hygro] vector and 0.5 μg pRL-TK vector (as an internal control). Six hours after transfection, cells were incubated in the presence of PMA (10 ng/ml) for 72 hrs. Then, the medium was replaced with fresh medium containing different concentrations of LPS as indicated and the cells were incubated for another 24 hrs, and subjected to luciferase assays. Relative luciferase activity is expressed as a fold of the luciferase activity of U937-SLC11A1Wt cells without treatment with LPS. The data shown (mean ± S.E.) are the averages of three independent experiments performed in triplicate. *P < 0.05, *P* < 0.01, ***P < 0.001, when compared with U937 cells expressing mutant SLC11A1. (C) U937-SLC11A1Wt or U937-SLC11A1Y15F cells were treated with PMA (10ng/ml) for 72 hrs, followed by treatment with various concentrations of LPS (0, 20, 40 and 80 ng/ml) for 24 hrs. Nuclear extracts were incubated with γ-^32^P-labeled wild type NF-κB oligonucleotides (Wt) or mutant NF-κB oligonucleotides (Mt). DNA-protein complexes were run on 4% non-denaturing polyacrylamide gels, followed by autoradiography.

To assess if tyrosine phosphorylation affects the function of SLC11A1, U937 cells stably expressing myc-tagged Wt SLC11A1,Y15F or Y38F mutant were differentiated into macrophages by treatment with PMA for 72 hrs. Then, the cells were treated with various concentrations of LPS (0, 20, 40 and 80 ng/ml) for another 24 hrs. NO concentrations were detected using Griess reagents. Compared with U937 cells expressing Wt SLC11A1, LPS-induced NO production was significantly decreased in the cells expressing the Y15F mutant but not in the cells expressing the Y38F mutant (Fig 8A) that exhibit a similar reduction in protein expression as observed in Y15F mutant-expressing cells but can still be phosphorylated (Fig 7). Previous studies have shown that NF-κB activation plays an important role in NO production [59–61]. SLC11A1 expression modulates LPS-stimulated NO production through regulating the activation and DNA binding of NF-κB [62]. Based on these findings, it is postulated that phosphorylation of SLC11A1 on Tyr-15 regulates the NF-κB activity, and then modulates NO production. To confirm this, U937 cells stably expressing Wt SLC11A1, Y15F or Y38F mutant were co-transfected with a firefly NF-κB luciferase reporter gene and a Renilla luciferase plasmid pRL-TK (as a control for transfection efficiency) and treated with LPS at different concentrations. As shown in Fig 8B, LPS-induced NF-κB transcriptional activity was significantly higher in U937-SLC11A1Wt cells compared with that in U937-SLC11A1Y15F cells at all three different concentrations, but there was no significant difference between SLC11A1 Wt- and Y38F mutant-expressing cells In consistent with this result, we also observed that LPS-induced DNA binding activity of NF-κB was reduced in U937 cells expressing SLC11A1Y15F mutant. No shift bands were observed when using mutant NF-κB oligonucletide as a probe, demonstrating a specific binding of NF-κB (Fig 8C). The specificity of the NF-κB binding was also confirmed by an EMSA using 100-fold excess of either unlabelled NF-κB oligonucleotide or mutant NF-κB oligonucleotide as a competitor (data not shown).

The expression level of SLC11A1 Y15F mutant was lower than that of Wt SLC11A1 in stably transfected cell lines (around 72–83% of Wt SLC11A1), therefore, lower LPS-induced NO production and NF-κB activation in Y15F mutant cells could be caused by the less expression of the SLC11A1 mutant. To exclude this possibility, the two mutant cell lines that stably express SLC11A1 Y15F or Y38F mutant were re-generated. We selected two stable cell clones that express mutant SLC11A1 (Y15F or Y38F) at almost the same level as the cell clone expressing Wt SLC11A1 (S2A Fig) and repeated above experiments. We observed again that LPS-induced NO production and NF-κB activation was impaired in the new generated Y15F mutant-expressing cells but not in the Y38F mutant-expressing cells (S2B and S2C Fig). Our results confirm that decrease in LPS-induced NO production and NF-κB activation in the Y15F mutant-expressing cells was not due to the less expression of the mutant SLC11A1. Overall, these results suggest that Tyr-15 phosphorylation is required for SLC11A1-mediated NF-κB activation and up-regulation of NO production induced by LPS.

## Discussion

Macrophages execute their antimicrobial activities by producing different biochemical mediators, including NO. When stimulated with lipopolysaccharide, interferon-γ, tumor necrosis factor-α or infected with several intracellular microorganisms, macrophages release NO due to the expression of inducible NO synthase (iNOS) [63]. The expression of iNOS is induced by the activated NF-κB at transcriptional level [64]. In unstimulated cells, the NF-κB is sequestered in the cytoplasm due to the association with its inhibitory protein, IκB. Macrophages activation by external stimuli results in the phosphorylation of IκB followed by ubquitination, which leads to the degradation of IκB and release of NF-κB. NF-κB is then translocated into the nucleus and induces the expression of target genes that have DNA-binding sites for it such as iNOS and interferon regulatory factor 1 [64]. NO production correlates with inhibition of intracellular microorganisms, such as *Mycobacterium* [65,66], *Toxoplasma* [67,68], *leishmania* [69,70] and *Listeria* [71,72] species. In the mouse, Slc11a1 gene has been shown to regulate the iNOS expression and NO production [73,74]. Mutation at Slc11a1 protein abrogrates its functions [75,76] and macrophages carrying mutant Slc11a1 produce significant less amount of nitric oxide in response to intracellular infection [73,77]. The growth inhibition of virulent *Mycobacterium tuberculosis* by macrophages carrying functional Slc11a1 can be abrogated by the addition of iNOS inhibitors, which suggests the importance of NO generation and its modulation by Slc11a1 for host defense against intracellular pathogens [78]. Our results revealed that human SLC11A1 is phosphorylated by c-Src kinase at Tyr15 and this type of phosphorylation is required for SLC11A1-mediated modulation of NF-κB activation and NO production induced by LPS in the differentiating macrophages. It has been shown that SLC11A1 is phosphorylated in macrophages and SLC11A1 phosphorylation is altered in response to cytokine stimulation [53,79]. It would be interesting to study further if the SLC11A1 phosphorylation is also required for SLC11A1-modulated NO production in response to other stimuli such as IFN-γ, and intracellular infection.

Phosphorylation of tyrosine residues by c-Src and/or other members of the SFKs has been found in a variety of proteins that are implicated in many important cellular processes, such as cell growth, differentiation, adhesion, transcription, ion channels, ROS production, as well as synaptic transmission and plasticity [80–84]. In this study, we provide evidence that SLC11A1 is phosphorylated by Src family kinases at tyrosine 15 present in a conserved tyrosine-based motif (YGSI) among all species. The phosphorylated ^15^YGSI^18^ motif resembles the SH2 domain binding motif pYXX (I/L/V), which can be recognized and bound by a class of SH2 domain-containing proteins. The SH2 domain was first identified in the oncoproteins Src and Fps and this domain is about 100 amino-acid residues long. Human genomic analysis revealed 121 SH2 domains that are distributed in 115 individual proteins involved in a wide range of signaling events [85]. SH2 domains typically bind a specific tyrosine phosphorylation site within a target protein and thereby linking activated protein tyrosine kinases to downstream signaling pathways that regulate gene expression and cellular activation processes. These include cell differentiation, cell migration and recognition of receptor tyrosine kinases [86]. It is postulated that a certain SH2 domain-containing protein is recruited to the pYGSI motif in response to LPS stimulation and controls the downstream events such as NF-kB signaling pathway and NO production. Identification of this SH2 domain-containing protein would help to understand the role of SLC11A1 in macrophage activation. In addition, as Src family kinases have been shown to be implicated in the macrophage inflammatory response to various stimuli [87], it will be interesting to investigate whether tyrosine 15 phosphorylation by Src family kinases is involved in SLC11A1-mediated regulation of other signal pathways and inflammatory gene expression.

In this study, we found that knockdown of c-Src kinase by siRNA results in a significant reduction in tyrosine phosphorylation of SLC11A1 compared with untreated cells. Further analysis showed that the level of tyrosine phosphorylation of SLC11A1 in c-Src knockdown cells was higher than that in the cells treated with PP2 (*P*<0.05), which might be caused by two reasons: (1) transfection of c-Src siRNA was unable to completely inhibit the c-Src expression; (2) c-Src, although important, may not be the only one of SFKs that is involved in the tyrosine phosphorylation of SLC11A1. So far, the Src family of protein-tyrosine kinases is composed of eleven members, which share major structural and regulatory features. Along with Src, Hck, Fyn and Fgr are also expressed in macrophages [88]. Gotoh and colleagues demonstrated that β-adducin is phosphorylated by Fyn at tyrosine 489, resulting in its recruitment to the Fyn-enriched region in the plasma membrane [89]. Radha and colleagues have shown that the guanine nucleotide exchange factor C3G is phosphorylated by HcK on tyrosine 504. Unlike C3G, which is mostly cytosolic, pY504-C3G translocates to the Golgi and subcortical cytoskeleton, indicating that Hck regulates C3G localization within the cell [90]. As Fyn and Hck kinases are also involved in protein phosphorylation, it remains to be established whether Fyn and Hck participate in the SLC11A1 phosphorylation.

In conclusion, we provide a strong evidence that c-Src kinase is involved in tyrosine phosphorylation of the SLC11A1 protein and affects the functions of SLC11A1 protein such as modulation of NF-κB activation and NO production.

## Supporting information

S1 Fig PMA-induced differentiation of HL-60 cells into macrophages. HL-60 cells were treated with 10 ng/ml PMA for 48 hrs. The cell differentiation was assessed by flow cytometric analysis of CD11b and CD14 expression, two markers of myeloid differentiation.
(TIF)

S2 Fig Tyr-15 to Phe substitution impaires SLC11A1-mediated NO production and NF-κB activation. U937 cells were stably transfected with constructed pCB6 vectors expressing c-Myc-tagged wild-type (U937-SLC11A1 Wt) or mutated SLC11A1 (U937-SLC11A1Y15F and U937-SLC11A1Y38F). (A) Cell lysates were prepared, and expression of Wt and mutant SLC11A1 was detected by Western blot analysis. β-actin was used as a loading control. (B) Same as Fig 8A. (C) Same as Fig 8B.
(TIF)

## References

[1] Cellier M, Shustik C, Dalton W, Rich E, Hu J, Malo D, et al Expression of the human NRAMP1 gene in professional primary phagocytes: studies in blood cells and in HL-60 promyelocytic leukemia. J Leukoc Biol. 1997; 61: 96–105. 9000542 10.1002/jlb.61.1.96

[2] Stober CB, Brode S, White JK, Popoff JF, Blackwell JM. Slc11a1, formerly Nramp1, is expressed in dendritic cells and influences major histocompatibility complex class II expression and antigen-presenting cell function. Infect Immun 75: 5059–5067. doi: 10.1128/IAI.00153-07 17620357 10.1128/IAI.00153-07 PMC2044529

[3] Gruenheid S, Pinner E, Desjardins M, Gros P. Natural resistance to infection with intracellular pathogens: the Nramp1 protein is recruited to the membrane of the phagosome. J Exp Med. 1997; 185: 717–730. 9034150 10.1084/jem.185.4.717 PMC2196151

[4] Cuellar-Mata P, Jabado N, Liu J, Furuya W, Finlay BB, Gros P, et al Nramp1 modifies the fusion of Salmonella typhimurium-containing vacuoles with cellular endomembranes in macrophages. J Biol Chem. 2002; 277: 2258–2265. doi: 10.1074/jbc.M105508200 11700301 10.1074/jbc.M105508200

[5] Govoni G, Canonne-Hergaux F, Pfeifer CG, Marcus SL, Mills SD, Hackam DJ, et al Functional expression of Nramp1 in vitro in the murine macrophage line RAW264.7. Infect Immun. 1999; 67: 2225–2232. 10225878 10.1128/iai.67.5.2225-2232.1999 PMC115961

[6] Searle S, Bright NA, Roach TI, Atkinson PG, Barton CH, Meloen RH, et al Localisation of Nramp1 in macrophages: modulation with activation and infection. J Cell Sci. 1998;111: 2855–2866. 9730978 10.1242/jcs.111.19.2855

[7] Hackam DJ, Rotstein OD, Zhang W, Gruenheid S, Gros P, Grinstein S. Host resistance to intracellular infection: mutation of natural resistance-associated macrophage protein 1 (Nramp1) impairs phagosomal acidification. J Exp Med. 1998; 188: 351–364. 9670047 10.1084/jem.188.2.351 PMC2212455

[8] Goswami T, Bhattacharjee A, Babal P, Searle S, Moore E, Li M, et al Natural-resistance-associated macrophage protein 1 is an H+/bivalent cation antiporter. Biochem J. 2001; 354: 511–519. 11237855 10.1042/0264-6021:3540511 PMC1221682

[9] Jabado N, Jankowski A, Dougaparsad S, Picard V, Grinstein S, Gros P. Natural resistance to intracellular infections: natural resistance-associated macrophage protein 1 (Nramp1) functions as a pH-dependent manganese transporter at the phagosomal membrane. J Exp Med. 2000; 192: 1237–1248. 11067873 10.1084/jem.192.9.1237 PMC2193348

[10] Kuhn DE, Baker BD, Lafuse WP, Zwilling BS. Differential iron transport into phagosomes isolated from the RAW264.7 macrophage cell lines transfected with Nramp1Gly169 or Nramp1Asp169. J Leukoc Biol. 1999; 66: 113–119. 10410998 10.1002/jlb.66.1.113

[11] Kuhn DE, Lafuse WP, Zwilling BS. Iron transport into mycobacterium avium-containing phagosomes from an Nramp1(Gly169)-transfected RAW264.7 macrophage cell line. J Leukoc Biol. 2001; 69: 43–49. 11200066

[12] Biggs TE, Baker ST, Botham MS, Dhital A, Barton CH, Perry VH. Nramp1 modulates iron homoeostasis in vivo and in vitro: evidence for a role in cellular iron release involving de-acidification of intracellular vesicles. Eur J Immunol. 2001; 31: 2060–2070. doi: 10.1002/1521-4141(200107)31:7<2060::AID-IMMU2060>3.0.CO;2-L 11449359 10.1002/1521-4141(200107)31:7<2060::aid-immu2060>3.0.co;2-l

[13] Soe-Lin S, Sheftel AD, Wasyluk B, Ponka P. Nramp1 equips macrophages for efficient iron recycling. Exp Hematol. 2008; 36: 929–937. doi: 10.1016/j.exphem.2008.02.013 18456389 10.1016/j.exphem.2008.02.013

[14] Soe-Lin S, Apte SS, Andriopoulos B Jr., Andrews MC, Schranzhofer M, Kahawita T, et al Nramp1 promotes efficient macrophage recycling of iron following erythrophagocytosis in vivo. Proc Natl Acad Sci U S A. 2009; 106: 5960–5965. doi: 10.1073/pnas.0900808106 19321419 10.1073/pnas.0900808106 PMC2667064

[15] Soe-Lin S, Apte SS, Mikhael MR, Kayembe LK, Nie G, Ponka P. Both Nramp1 and DMT1 are necessary for efficient macrophage iron recycling. Exp Hematol. 2010; 38: 609–617. doi: 10.1016/j.exphem.2010.04.003 20394798 10.1016/j.exphem.2010.04.003

[16] Blackwell JM, Searle S, Mohamed H, White JK. Divalent cation transport and susceptibility to infectious and autoimmune disease: continuation of the Ity/Lsh/Bcg/Nramp1/Slc11a1 gene story. Immunol Lett. 2003; 85: 197–203. 12527228 10.1016/s0165-2478(02)00231-6

[17] Forbes JR, Gros P. Divalent-metal transport by NRAMP proteins at the interface of host-pathogen interactions. Trends Microbiol. 2001; 9: 397–403. 11514223 10.1016/s0966-842x(01)02098-4

[18] Fritsche G, Dlaska M, Barton H, Theurl I, Garimorth K, Weiss G. Nramp1 functionality increases inducible nitric oxide synthase transcription via stimulation of IFN regulatory factor 1 expression. J Immunol. 2003; 171: 1994–1998. 12902503 10.4049/jimmunol.171.4.1994

[19] Nevo Y, Nelson N. The NRAMP family of metal-ion transporters. Biochim Biophys Acta. 2006; 1763: 609–620. doi: 10.1016/j.bbamcr.2006.05.007 16908340 10.1016/j.bbamcr.2006.05.007

[20] Valdez Y, Grassl GA, Guttman JA, Coburn B, Gros P, Vallance BA, et al Nramp1 drives an accelerated inflammatory response during Salmonella-induced colitis in mice. Cell Microbiol. 2009; 11: 351–362. doi: 10.1111/j.1462-5822.2008.01258.x 19016783 10.1111/j.1462-5822.2008.01258.x

[21] Wojciechowski W, DeSanctis J, Skamene E, Radzioch D. Attenuation of MHC class II expression in macrophages infected with Mycobacterium bovis bacillus Calmette-Guerin involves class II transactivator and depends on the Nramp1 gene. J Immunol. 1999; 163: 2688–2696. 10453010

[22] Zwilling BS, Vespa L, Massie M. Regulation of I-A expression by murine peritoneal macrophages: differences linked to the Bcg gene. J Immunol. 1987; 138: 1372–1376. 3543128

[23] Nairz M, Fritsche G, Crouch ML, Barton HC, Fang FC, Weiss G. Slc11a1 limits intracellular growth of Salmonella enterica sv. Typhimurium by promoting macrophage immune effector functions and impairing bacterial iron acquisition. Cell Microbiol. 2009; 11: 1365–1381. doi: 10.1111/j.1462-5822.2009.01337.x 19500110 10.1111/j.1462-5822.2009.01337.x PMC3467104

[24] Gomez MA, Li S, Tremblay ML, Olivier M. NRAMP-1 expression modulates protein-tyrosine phosphatase activity in macrophages: impact on host cell signaling and functions. J Biol Chem. 2007;282: 36190–36198. doi: 10.1074/jbc.M703140200 17942403 10.1074/jbc.M703140200

[25] Hedges JF, Kimmel E, Snyder DT, Jerome M, Jutila MA. Solute Carrier 11A1 Is Expressed by Innate Lymphocytes and Augments Their Activation. J Immunol. 2013; 190: 4263–4273. doi: 10.4049/jimmunol.1200732 23509347 10.4049/jimmunol.1200732 PMC3622125

[26] Archer NS, Nassif NT, O'Brien BA. Genetic variants of SLC11A1 are associated with both autoimmune and infectious diseases: systematic review and meta-analysis. Genes Immun. 2015; 16: 275–283. doi: 10.1038/gene.2015.8 25856512 10.1038/gene.2015.8

[27] Nino-Moreno P, Turrubiartes-Martinez E, Oceguera-Maldonado B, Baltazar-Benitez N, Negrete-Gonzalez C, Oliva-Ramirez B, et al The Role of NRAMP1/SLC11A1 Gene Variant D543N (1730G/A) in the Genetic Susceptibility to Develop Rheumatoid Arthritis in the Mexican Mestizo population. Rev Invest Clin. 2017; 69: 5–10. 28239176 10.24875/ric.17002152

[28] Lin X, Hamilton-Williams EE, Rainbow DB, Hunter KM, Dai YD, Cheung J, etal Genetic interactions among Idd3, Idd5.1, Idd5.2, and Idd5.3 protective loci in the nonobese diabetic mouse model of type 1 diabetes. J Immunol. 2013; 190: 3109–3120. doi: 10.4049/jimmunol.1203422 23427248 10.4049/jimmunol.1203422 PMC3608810

[29] Akcakaya P, Azeroglu B, Even I, Ates O, Turker H, Ongen G, et al The functional SLC11A1 gene polymorphisms are associated with sarcoidosis in Turkish population. Mol Biol Rep. 2012; 39: 5009–5016. doi: 10.1007/s11033-011-1297-x 22160516 10.1007/s11033-011-1297-x

[30] Ates O, Dalyan L, Hatemi G, Hamuryudan V, Topal-Sarikaya A. Genetic susceptibility to Behcet's syndrome is associated with NRAMP1 (SLC11A1) polymorphism in Turkish patients. Rheumatol Int. 2009; 29: 787–791. doi: 10.1007/s00296-008-0763-9 18998137 10.1007/s00296-008-0763-9

[31] Ates O, Kurt S, Bozkurt N, Karaer H. NRAMP1 (SLC11A1) variants: genetic susceptibility to multiple Sclerosis. J Clin Immunol. 2010; 30: 583–586. doi: 10.1007/s10875-010-9422-5 20405176 10.1007/s10875-010-9422-5

[32] Smit JJ, van LH, Hoekstra MO, Nijkamp FP, Bloksma N. Influence of the macrophage bacterial resistance gene, Nramp1 (Slc11a1), on the induction of allergic asthma in the mouse. FASEB J. 2003; 17: 958–960. doi: 10.1096/fj.02-0985fje 12670871 10.1096/fj.02-0985fje

[33] Canhamero T, Reines B, Peters LC, Borrego A, Carneiro PS, Albuquerque LL, et al Distinct early inflammatory events during ear tissue regeneration in mice selected for high inflammation bearing Slc11a1 R and S alleles. Inflammation. 2011; 34: 303–313. doi: 10.1007/s10753-010-9235-y 20665098 10.1007/s10753-010-9235-y

[34] Barton CH, White JK, Roach TI, Blackwell JM. NH2-terminal sequence of macrophage-expressed natural resistance-associated macrophage protein (Nramp) encodes a proline/serine-rich putative Src homology 3-binding domain. J Exp Med. 1994; 179: 1683–1687. 7513015 10.1084/jem.179.5.1683 PMC2191468

[35] Gout I, Dhand R, Hiles ID, Fry MJ, Panayotou G, Das P, et al The GTPase dynamin binds to and is activated by a subset of SH3 domains. Cell. 1993; 75: 25–36. 8402898

[36] Kaneko T, Li L, Li SS. The SH3 domain—a family of versatile peptide- and protein-recognition module. Front Biosci. 2008; 13:4938–52. 18508559 10.2741/3053

[37] Kay BK, Williamson MP, Sudol M. The importance of being proline: the interaction of proline-rich motifs in signaling proteins with their cognate domains. FASEB J. 2000; 14: 231–241. 10657980

[38] Mayer BJ.SH3 domains: complexity in moderation. J Cell Sci. 2001; 114: 1253–1263. 11256992 10.1242/jcs.114.7.1253

[39] Yu H, Chen JK, Feng S, Dalgarno DC, Brauer AW, Schreiber SL. Structural basis for the binding of proline-rich peptides to SH3 domains. Cell. 1994; 76: 933–945. 7510218 10.1016/0092-8674(94)90367-0

[40] Aitio O, Hellman M, Kazlauskas A, Vingadassalom DF, Leong JM, Saksela K, et al Recognition of tandem PxxP motifs as a unique Src homology 3-binding mode triggers pathogen-driven actin assembly. Proc Natl Acad Sci U S A. 2010; 107: 21743–21748. doi: 10.1073/pnas.1010243107 21098279 10.1073/pnas.1010243107 PMC3003032

[41] Dalgarno DC, Botfield MC, Rickles RJ. SH3 domains and drug design: ligands, structure, and biological function. Biopolymers. 1997; 43: 383–400. doi: 10.1002/(SICI)1097-0282(1997)43:5<383::AID-BIP4>3.0.CO;2-R 10.1002/(SICI)1097-0282(1997)43:5<383::AID-BIP4>3.0.CO;2-R 9566119

[42] Mayer BJ, Baltimore D. Signalling through SH2 and SH3 domains. Trends Cell Biol. 1993; 3: 8–13. 14731533 10.1016/0962-8924(93)90194-6

[43] Pawson T. Protein modules and signalling networks. Nature. 1995; 373: 573–580. doi: 10.1038/373573a0 7531822 10.1038/373573a0

[44] Sen B, Johnson FM. Regulation of SRC family kinases in human cancers. J Signal Transduct 2011:865819 doi: 10.1155/2011/865819 Epub;%2011 Apr 4.: 865819. 21776389 10.1155/2011/865819 PMC3135246

[45] Geahlen RL, Handley MD, Harrison ML. Molecular interdiction of Src-family kinase signaling in hematopoietic cells. Oncogene. 2004; 23: 8024–8032. doi: 10.1038/sj.onc.1208078 15489920 10.1038/sj.onc.1208078

[46] Brunton VG, Ozanne BW, Paraskeva C, Frame MC. A role for epidermal growth factor receptor, c-Src and focal adhesion kinase in an in vitro model for the progression of colon cancer. Oncogene. 1997;14: 283–293. doi: 10.1038/sj.onc.1200827 9018114 10.1038/sj.onc.1200827

[47] Edwards JC, Cohen C, Xu W, Schlesinger PH. c-Src control of chloride channel support for osteoclast HCl transport and bone resorption. J Biol Chem. 2006;281: 28011–28022. doi: 10.1074/jbc.M605865200 16831863 10.1074/jbc.M605865200 PMC1808340

[48] Fleming RY, Ellis LM, Parikh NU, Liu W, Staley CA, Gallick GE. Regulation of vascular endothelial growth factor expression in human colon carcinoma cells by activity of src kinase. Surgery. 1997;122: 501–507. 9288158 10.1016/s0039-6060(97)90044-1

[49] Holmes TC, Fadool DA, Ren R, Levitan IB. Association of Src tyrosine kinase with a human potassium channel mediated by SH3 domain. Science. 1996;274: 2089–2091. 8953041 10.1126/science.274.5295.2089

[50] Owens DW, McLean GW, Wyke AW, Paraskeva C, Parkinson EK, Frame MC, et al The catalytic activity of the Src family kinases is required to disrupt cadherin-dependent cell-cell contacts. Mol Biol Cell. 2000;11: 51–64. 10637290 10.1091/mbc.11.1.51 PMC14756

[51] Blom N, Gammeltoft S, Brunak S. Sequence and structure-based prediction of eukaryotic protein phosphorylation sites. J Mol Biol. 1999;294: 1351–1362. doi: 10.1006/jmbi.1999.3310 10600390 10.1006/jmbi.1999.3310

[52] Xue Y, Ren J, Gao X, Jin C, Wen L, Yao X. GPS 2.0, a tool to predict kinase-specific phosphorylation sites in hierarchy. Mol Cell Proteomics. 2008;7: 1598–1608. doi: 10.1074/mcp.M700574-MCP200 18463090 10.1074/mcp.M700574-MCP200 PMC2528073

[53] Barton CH, Biggs TE, Baker ST, Bowen H, Atkinson PG. Nramp1: a link between intracellular iron transport and innate resistance to intracellular pathogens. J Leukoc Biol. 1999;66: 757–762. 10577506 10.1002/jlb.66.5.757

[54] Liu J, Liao Z, Camden J, Griffin KD, Garrad RC, Santiago-Perez LI, et al Src homology 3 binding sites in the P2Y2 nucleotide receptor interact with Src and regulate activities of Src, proline-rich tyrosine kinase 2, and growth factor receptors. J Biol Chem. 2004;279: 8212–8218. doi: 10.1074/jbc.M312230200 14670955 10.1074/jbc.M312230200

[55] Canonne-Hergaux F, Calafat J, Richer E, Cellier M, Grinstein S, Borregaard N, et al Expression and subcellular localization of NRAMP1 in human neutrophil granules. Blood. 2002;100: 268–275. 12070036 10.1182/blood.v100.1.268

[56] Xu YZ, Di MS, Gallouzi I, Rola-Pleszczynski M, Radzioch D. RNA-binding protein HuR is required for stabilization of SLC11A1 mRNA and SLC11A1 protein expression. Mol Cell Biol. 2005;25: 8139–8149. doi: 10.1128/MCB.25.18.8139-8149.2005 16135804 10.1128/MCB.25.18.8139-8149.2005 PMC1234318

[57] Chen F, Kuhn DC, Gaydos LJ, Demers LM. Induction of nitric oxide and nitric oxide synthase mRNA by silica and lipopolysaccharide in PMA-primed THP-1 cells. APMIS. 1996;104: 176–182. 8611191 10.1111/j.1699-0463.1996.tb00705.x

[58] Abram CL, Courtneidge SA. Src family tyrosine kinases and growth factor signaling. Exp Cell Res. 2000;254: 1–13. doi: 10.1006/excr.1999.4732 10623460 10.1006/excr.1999.4732

[59] Arias-Salvatierra D, Silbergeld EK, Acosta-Saavedra LC, Calderon-Aranda ES. Role of nitric oxide produced by iNOS through NF-kappaB pathway in migration of cerebellar granule neurons induced by Lipopolysaccharide. Cell Signal. 2011;23: 425–435. doi: 10.1016/j.cellsig.2010.10.017 20955790 10.1016/j.cellsig.2010.10.017

[60] Jalal DI, Kone BC. Src activation of NF-kappaB augments IL-1beta-induced nitric oxide production in mesangial cells. J Am Soc Nephrol. 2006; 17: 99–106. doi: 10.1681/ASN.2005070693 16338964 10.1681/ASN.2005070693

[61] Jones E, Adcock IM, Ahmed BY, Punchard NA. Modulation of LPS stimulated NF-kappaB mediated Nitric Oxide production by PKCepsilon and JAK2 in RAW macrophages. J Inflamm (Lond). 2007;4:23.: 23. 18036230 10.1186/1476-9255-4-23 PMC2211292

[62] Gomez MA, Li S, Tremblay ML, Olivier M. NRAMP-1 expression modulates protein-tyrosine phosphatase activity in macrophages: impact on host cell signaling and functions. J Biol Chem. 2007;282: 36190–36198. doi: 10.1074/jbc.M703140200 17942403 10.1074/jbc.M703140200

[63] Green SJ, Nacy CA. Antimicrobial and immunopathologic effects of cytokine-induced nitric oxide synthesis. Curr Opin Infect Dis. 1993;6: 384–396.

[64] Xie QW, Kashiwabara Y, Nathan C. Role of transcription factor NF-kappa B/Rel in induction of nitric oxide synthase. J Biol Chem. 1994;269: 4705–4708. 7508926

[65] Flesch IE, Kaufmann SH. Mechanisms involved in mycobacterial growth inhibition by gamma interferon-activated bone marrow macrophages: role of reactive nitrogen intermediates. Infect Immun. 1991;59: 3213–3218. 1908829 10.1128/iai.59.9.3213-3218.1991 PMC258155

[66] Chan J, Tanaka K, Carroll D, Flynn J, Bloom BR. Effects of nitric oxide synthase inhibitors on murine infection with Mycobacterium tuberculosis. Infect Immun. 1995;63: 736–740. 7529749 10.1128/iai.63.2.736-740.1995 PMC173063

[67] Hayashi S, Chan CC, Gazzinelli RT, Pham NT, Cheung MK, Roberge FG. Protective role of nitric oxide in ocular toxoplasmosis. Br J Ophthalmol. 1996;80: 644–648. 8795379 10.1136/bjo.80.7.644 PMC505561

[68] Authier H, Cassaing S, Bans V, Batigne P, Bessieres MH, Pipy B. IL-13 pre-treatment of murine peritoneal macrophages increases their anti-Toxoplasma gondii activity induced by lipopolysaccharides. Int J Parasitol. 2008;38: 341–352. doi: 10.1016/j.ijpara.2007.08.002 17923133 10.1016/j.ijpara.2007.08.002

[69] Assreuy J, Cunha FQ, Epperlein M, Noronha-Dutra A, O'Donnell CA, Liew FY, et al Production of nitric oxide and superoxide by activated macrophages and killing of Leishmania major. Eur J Immunol. 1994;24: 672–676. doi: 10.1002/eji.1830240328 8125136 10.1002/eji.1830240328

[70] Winberg ME, Rasmusson B, Sundqvist T. Leishmania donovani: inhibition of phagosomal maturation is rescued by nitric oxide in macrophages. Exp Parasitol. 2007;117: 165–170. doi: 10.1016/j.exppara.2007.04.004 17511987 10.1016/j.exppara.2007.04.004

[71] Boockvar KS, Granger DL, Poston RM, Maybodi M, Washington MK, Hibbs JB Jr., et al Nitric oxide produced during murine listeriosis is protective. Infect Immun. 1994;62: 1089–1100. 7509315 10.1128/iai.62.3.1089-1100.1994 PMC186228

[72] Remer KA, Jungi TW, Fatzer R, Tauber MG, Leib SL. Nitric oxide is protective in listeric meningoencephalitis of rats. Infect Immun. 2001;69: 4086–4093. doi: 10.1128/IAI.69.6.4086-4093.2001 11349080 10.1128/IAI.69.6.4086-4093.2001 PMC98473

[73] Barrera LF, Kramnik I, Skamene E, Radzioch D. Nitrite production by macrophages derived from BCG-resistant and -susceptible congenic mouse strains in response to IFN-gamma and infection with BCG. Immunology. 1994;82: 457–464. 7959883 PMC1414881

[74] Barton CH, Whitehead SH, Blackwell JM. Nramp transfection transfers Ity/Lsh/Bcg-related pleiotropic effects on macrophage activation: influence on oxidative burst and nitric oxide pathways. Mol Med. 1995;1: 267–279. 8529105 PMC2229912

[75] Malo D, Vogan K, Vidal S, Hu J, Cellier M, Schurr E, et al Haplotype mapping and sequence analysis of the mouse Nramp gene predict susceptibility to infection with intracellular parasites. Genomics. 1994;23: 51–61. doi: 10.1006/geno.1994.1458 7829102 10.1006/geno.1994.1458

[76] Vidal S, Tremblay ML, Govoni G, Gauthier S, Sebastiani G, Malo D, et al The Ity/Lsh/Bcg locus: natural resistance to infection with intracellular parasites is abrogated by disruption of the Nramp1 gene. J Exp Med. 1995;182: 655–666. 7650477 10.1084/jem.182.3.655 PMC2192162

[77] Formica S, Roach TI, Blackwell JM. Interaction with extracellular matrix proteins influences Lsh/Ity/Bcg (candidate Nramp) gene regulation of macrophage priming/activation for tumour necrosis factor-alpha and nitrite release. Immunology. 1994;82: 42–50. 8045593 PMC1414848

[78] Arias M, Rojas M, Zabaleta J, Rodriguez JI, Paris SC, Barrera LF, et al Inhibition of virulent Mycobacterium tuberculosis by Bcg(r) and Bcg(s) macrophages correlates with nitric oxide production. J Infect Dis. 1997;176: 1552–1558. 9395367 10.1086/514154

[79] Vidal SM, Pinner E, Lepage P, Gauthier S, Gros P. Natural resistance to intracellular infections: Nramp1 encodes a membrane phosphoglycoprotein absent in macrophages from susceptible (Nramp1 D169) mouse strains. J Immunol. 1996;157: 3559–3568. 8871656

[80] Wang GZ, Goff SP. Regulation of Yin Yang 1 by Tyrosine Phosphorylation. J Biol Chem. 2015;290: 21890–21900. doi: 10.1074/jbc.M115.660621 26198631 10.1074/jbc.M115.660621 PMC4571944

[81] Lahdenpera J, Kilpelainen P, Liu XL, Pikkarainen T, Reponen P, Ruotsalainen V, et al Clustering-induced tyrosine phosphorylation of nephrin by Src family kinases. Kidney Int. 2003;64: 404–413. doi: 10.1046/j.1523-1755.2003.00097.x 12846735 10.1046/j.1523-1755.2003.00097.x

[82] Beacham D, Ahn M, Catterall WA, Scheuer T. Sites and molecular mechanisms of modulation of Na(v)1.2 channels by Fyn tyrosine kinase. J Neurosci. 2007;27: 11543–11551. doi: 10.1523/JNEUROSCI.1743-07.2007 17959797 10.1523/JNEUROSCI.1743-07.2007 PMC6673227

[83] Ogura M, Yamaki J, Homma MK, Homma Y. Phosphorylation of flotillin-1 by mitochondrial c-Src is required to prevent the production of reactive oxygen species. FEBS Lett. 2014;588: 2837–2843. doi: 10.1016/j.febslet.2014.06.044 24983503 10.1016/j.febslet.2014.06.044

[84] Wu HC, Chang CH, Peng HY, Chen GD, Lai CY, Hsieh MC, et al EphrinB2 induces pelvic-urethra reflex potentiation via Src kinase-dependent tyrosine phosphorylation of NR2B. Am J Physiol Renal Physiol. 2011;300: F403–F411. doi: 10.1152/ajprenal.00520.2010 21147838 10.1152/ajprenal.00520.2010

[85] Liu BA, Jablonowski K, Raina M, Arce M, Pawson T, Nash PD. The human and mouse complement of SH2 domain proteins-establishing the boundaries of phosphotyrosine signaling. Mol Cell. 2006;22: 851–868. doi: 10.1016/j.molcel.2006.06.001 16793553 10.1016/j.molcel.2006.06.001

[86] Liu BA, Engelmann BW, Nash PD. The language of SH2 domain interactions defines phosphotyrosine-mediated signal transduction. FEBS Lett. 2012;586: 2597–2605. doi: 10.1016/j.febslet.2012.04.054 22569091 10.1016/j.febslet.2012.04.054

[87] Freudenburg W, Buller RM, Corbett JA. Src family kinases participate in the regulation of encephalomyocarditis virus-induced cyclooxygenase-2 expression by macrophages. J Gen Virol. 2010;91: 2278–2285. doi: 10.1099/vir.0.022665-0 20505008 10.1099/vir.0.022665-0 PMC3052521

[88] Ziegler SF, Wilson CB, Perlmutter RM. Augmented expression of a myeloid-specific protein tyrosine kinase gene (hck) after macrophage activation. J Exp Med. 1988;168: 1801–1810. 3141554 10.1084/jem.168.5.1801 PMC2189116

[89] Gotoh H, Okumura N, Yagi T, Okumura A, Shima T, Nagai K. Fyn-induced phosphorylation of beta-adducin at tyrosine 489 and its role in their subcellular localization. Biochem Biophys Res Commun. 2006;346: 600–605. doi: 10.1016/j.bbrc.2006.05.167 16765915 10.1016/j.bbrc.2006.05.167

[90] Radha V, Rajanna A, Swarup G. Phosphorylated guanine nucleotide exchange factor C3G, induced by pervanadate and Src family kinases localizes to the Golgi and subcortical actin cytoskeleton. BMC Cell Biol. 2004;5:31 doi: 10.1186/1471-2121-5-31 15320955 10.1186/1471-2121-5-31 PMC515295

