# Supplementary figures and images for: c-Src kinase is involved in the tyrosine phosphorylation and activity of SLC11A1 in differentiating macrophages
Source: PLoS One. 2018 May 3;13(5):e0196230. doi: 10.1371/journal.pone.0196230 (PMC5933793; doi:10.1371/journal.pone.0196230)

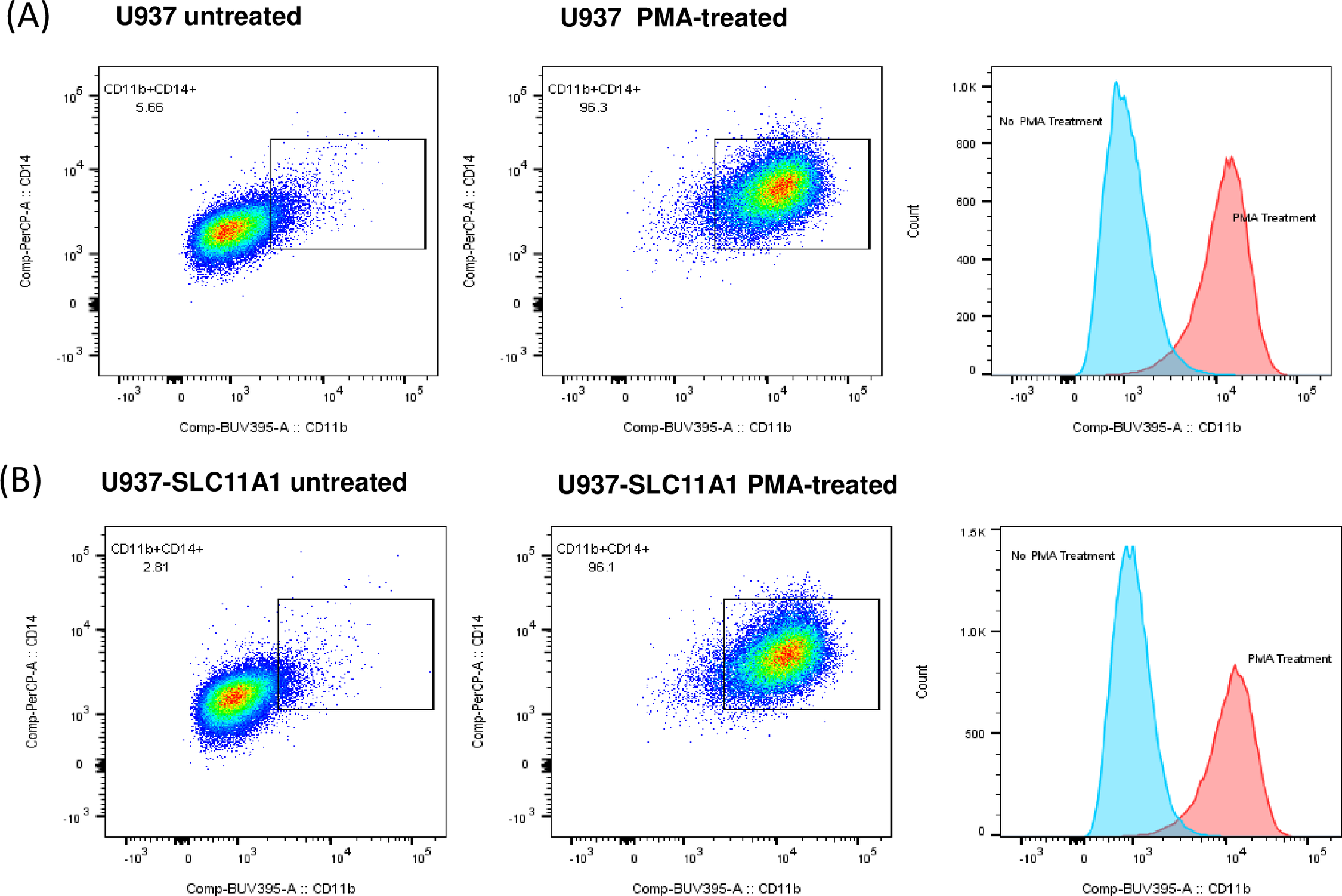

Supplement: S1 Fig — HL-60 cells were treated with 10 ng/ml PMA for 48 hrs. The cell differentiation was assessed by flow cytometric analysis of CD11b and CD14 expression, two markers of myeloid differentiation. (TIF) [file pone.0196230.s001.tif]

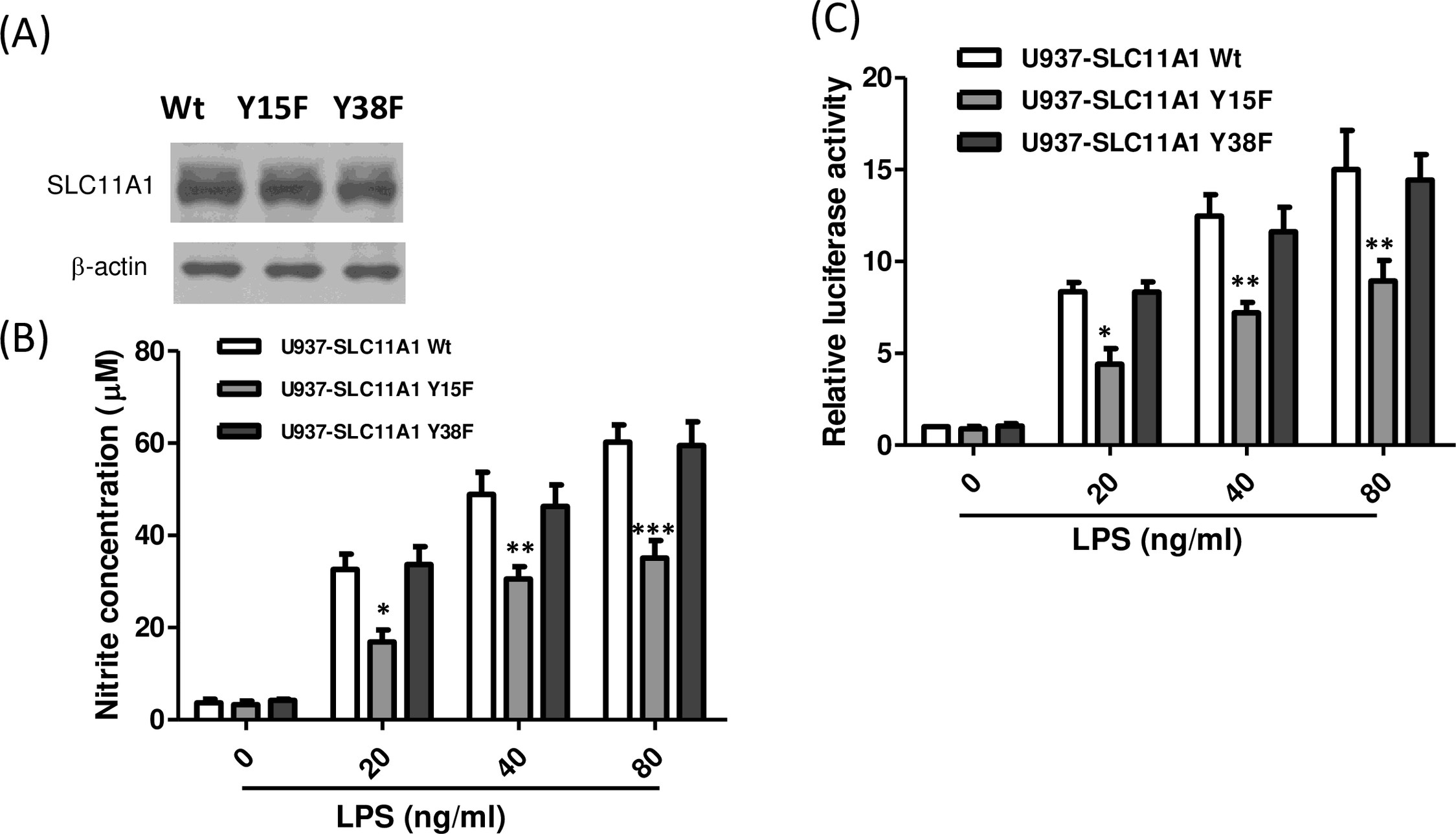

Supplement: S2 Fig — U937 cells were stably transfected with constructed pCB6 vectors expressing c-Myc-tagged wild-type (U937-SLC11A1 Wt) or mutated SLC11A1 (U937-SLC11A1Y15F and U937-SLC11A1Y38F). (A) Cell lysates were prepared, and expression of Wt and mutant SLC11A1 was detected by Western blot analysis. β-actin was used as a loading control. (B) Same as Fig 8A. (C) Same as Fig 8B. (TIF) [file pone.0196230.s002.tif]
